# The lipidomics reporting checklist a framework for transparency of lipidomic experiments and repurposing resource data

**DOI:** 10.1016/j.jlr.2024.100621

**Published:** 2024-08-14

**Authors:** Dominik Kopczynski, Christer S. Ejsing, Jeffrey G. McDonald, Takeshi Bamba, Erin S. Baker, Justine Bertrand-Michel, Britta Brügger, Cristina Coman, Shane R. Ellis, Timothy J. Garrett, William J. Griffiths, Xue Li Guan, Xianlin Han, Marcus Höring, Michal Holčapek, Nils Hoffmann, Kevin Huynh, Rainer Lehmann, Jace W. Jones, Rima Kaddurah-Daouk, Harald C. Köfeler, Peter J. Meikle, Thomas O. Metz, Valerie B. O’Donnell, Daisuke Saigusa, Dominik Schwudke, Andrej Shevchenko, Federico Torta, Juan Antonio Vizcaíno, Ruth Welti, Markus R. Wenk, Denise Wolrab, Yu Xia, Kim Ekroos, Robert Ahrends, Gerhard Liebisch

**Affiliations:** 1 Department of Analytical Chemistry, Faculty of Chemistry, University of Vienna, Vienna, Austria; 2 Department of Biochemistry and Molecular Biology, VILLUM Center for Bioanalytical Sciences, University of Southern Denmark, Odense, Denmark; 3 Cell Biology and Biophysics Unit, European Molecular Biology Laboratory, Heidelberg, Germany; 4 Center for Human Nutrition and Department of Molecular Genetics, UT Southwestern Medical Center, Dallas, Texas, USA; 5 Division of Metabolomics/Mass Spectrometry Center, Medical Research Center for High Depth Omics, Medical Institute of Bioregulation, Kyushu University, Fukuoka, Japan; 6 Department of Chemistry, University of North Carolina at Chapel Hill, Chapel Hill, North Carolina, USA; 7 MetaboHUB-Metatoul, National Infrastructure of Metabolomics and Fluxomics, Inserm I2MC, Toulouse, France; 8 Heidelberg University Biochemistry Center (BZH), University of Heidelberg, Heidelberg, Germany; 9 Molecular Horizons and School of Chemistry and Molecular Bioscience, University of Wollongong, Wollongong, NSW, Australia; 10 Department of Pathology, Immunology and Laboratory Medicine, University of Florida, Gainesville, Florida, USA; 11 Swansea University Medical School, Swansea, Wales, UK; 12 Lee Kong Chian School of Medicine, Nanyang Technological University, Singapore, Singapore; 13 Barshop Institute for Longevity and Aging Studies, University of Texas Health Science Center at San Antonio, San Antonio, Texas, USA; 14 Institute of Clinical Chemistry and Laboratory Medicine, University of Regensburg, Regensburg, Germany; 15 Department of Analytical Chemistry, Faculty of Chemical Technology, University of Pardubice, Pardubice, Czech Republic; 16 Institute for Bio- and Geosciences (IBG-5), Forschungszentrum Jülich GmbH, Jülich, Germany; 17 Baker Heart and Diabetes Institute, Melbourne, VIC, Australia; 18 Department of Cardiovascular Research Translation and Implementation, La Trobe University, Bundoora, VIC, Australia; 19 Institute for Clinical Chemistry and Pathobiochemistry, Department for Diagnostic Laboratory Medicine, University Hospital Tuebingen, Tuebingen, Germany; 20 Department of Pharmaceutical Sciences, School of Pharmacy, University of Maryland, Baltimore, Maryland, USA; 21 Department of Psychiatry and Behavioural Sciences, Duke University, Durham, North Carolina, USA; 22 Duke Institute of Brain Sciences, Duke University, Durham, North Carolina, USA; 23 Department of Medicine, Duke University, Durham, North Carolina, USA; 24 Core Facility Mass Spectrometry and Lipidomics, ZMF, Medical University of Graz, Graz, Austria; 25 Biological Sciences Division, Pacific Northwest National Laboratory, Richland, Washington, USA; 26 Systems Immunity Research Institute, School of Medicine, Cardiff University, Cardiff, UK; 27 Laboratory of Biomedical and Analytical Sciences, Faculty of Pharma-Science, Teikyo University, Tokyo, Japan; 28 Division of Bioanalytical Chemistry, Research Center Borstel – Leibniz Lung Center, Borstel, Germany; 29 German Center for Infection Research, Thematic Translational Unit Tuberculosis, Partner Site Hamburg-Lübeck-Borstel-Riems, Germany; 30 German Center for Lung Research (DZL), Airway Research Center North (ARCN), Borstel, Germany; 31 Max-Planck-Institute of Molecular Cell Biology and Genetics, Dresden, Germany; 32 Singapore Lipidomics Incubator (SLING), Department of Biochemistry, YLL School of Medicine, National University of Singapore, Singapore, Singapore; 33 Signature Research Program in Cardiovascular and Metabolic Disorders, Duke-NUS Medical School, Singapore, Singapore; 34 European Molecular Biology Laboratory, European Bioinformatics Institute (EMBL-EBI), Wellcome Trust Genome Campus, Hinxton, Cambridge, UK; 35 Kansas Lipidomics Research Center, Division of Biology, Kansas State University, Manhattan, Kansas, USA; 36 MOE Key Laboratory of Bioorganic Phosphorus Chemistry & Chemical Biology, Department of Chemistry, Tsinghua University, Beijing, China; 37 Lipidomics Consulting Ltd., Esbo, Finland

**Keywords:** checklist, lipid metabolism, lipidomics, mass spectrometry, metabolomics, reference standards, FAIR, quality control

## Abstract

The rapid increase in lipidomic studies has led to a collaborative effort within the community to establish standards and criteria for producing, documenting, and disseminating data. Creating a dynamic easy-to-use checklist that condenses key information about lipidomic experiments into common terminology will enhance the field's consistency, comparability, and repeatability. Here, we describe the structure and rationale of the established Lipidomics Minimal Reporting Checklist to increase transparency in lipidomics research.

A rapidly growing interest in lipidomics necessitates harmonizing experimental data reporting, including both metadata and raw/actual data, to ensure transparency, comparability, repurposing, and confidence in lipidomics data (1). Furthermore, the complexity and nuances of lipidomics require detailed experimental settings to enable researchers to reproduce and assess the applicability of reported methods. In lipidomics, data processing is a critical component of the process, which includes lipid identification and quantification using mass spectrometry alone or in combination with chromatography. This is especially important in the context of automated lipid annotation by search algorithms, as they may incorrectly annotate and interpret experimental data (2).

The community is making efforts to standardize lipid nomenclature (3) and raise awareness of the capabilities of different mass spectrometry platforms for lipid structural elucidation (4). These steps are important for achieving 'Findable, Accessible, Interoperable, and Reusable' (FAIR) data (5). However, to achieve FAIR data reporting, in principle, a standardized reporting of the entire lipidomics workflow is required, describing the samples analyzed, experimental and data analysis methods as well as quality control and reporting of the data. The recently introduced Lipidomics Minimal Reporting Checklist (6) helps to close this gap in implementing the FAIR principles in lipidomics research, addressing mainly interoperability and reusability. The checklist's scope is described below, along with an explanation of how this information will be utilized to fulfill the FAIR principles and ensure the quality of lipidomic datasets. Importantly, the use of the checklist is currently limited to mass spectrometry-based analysis and should not be limited to lipidomics workflows, but can also include metabolomics workflows covering lipid molecules.

## Aim and concept of the reporting checklist

**Fig. 1:**
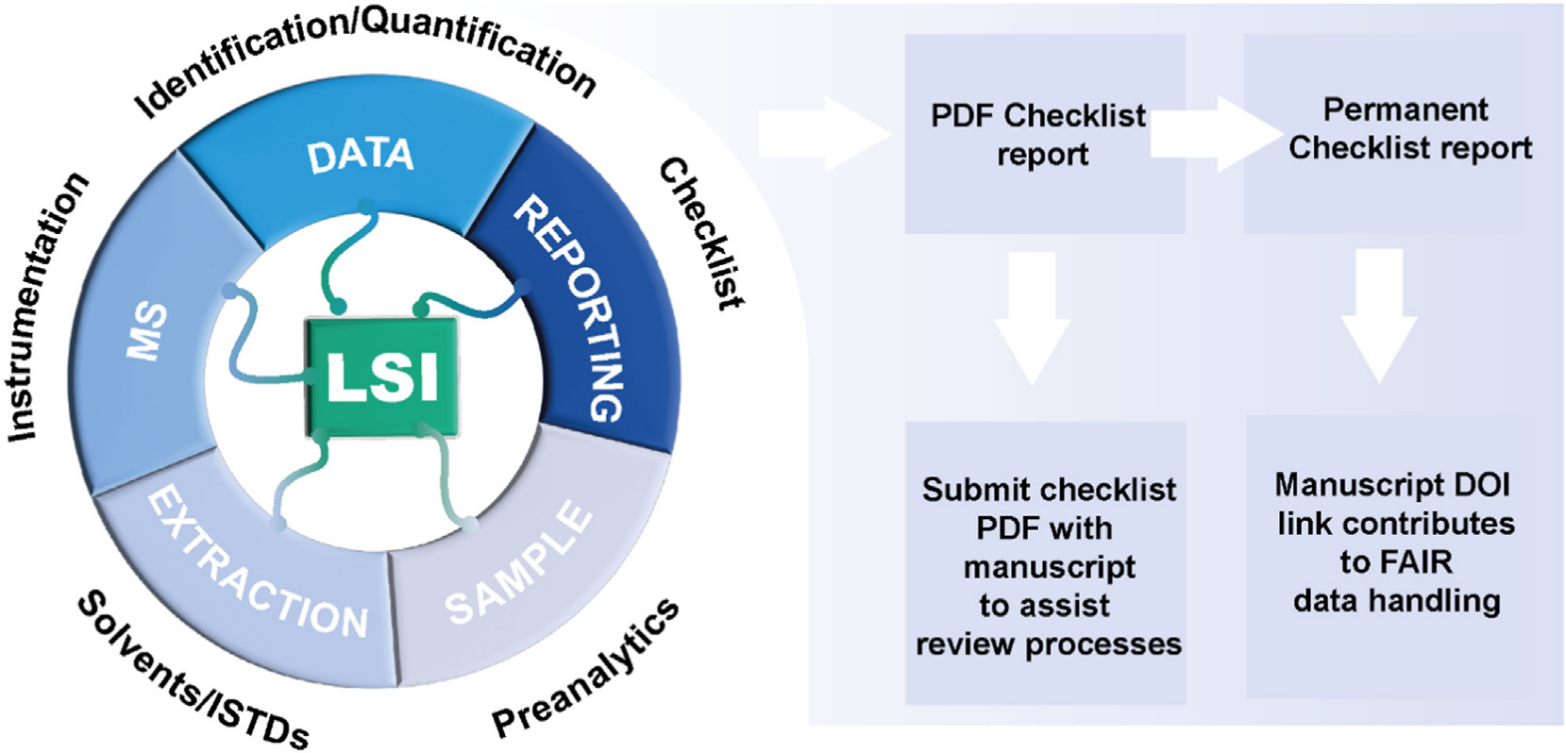
Scope and aim of the lipidomics reporting checklist.

The Lipidomics Minimal Reporting Checklist (6) has been established and is continuously curated by the Lipidomics Standard Initiative (LSI, https://lipidomicstandards.org/) (1), an interest group affiliated with the International Lipidomics Society (ILS, https://lipidomicssociety.org/). The checklist (version 2.4.0) is based on consensus-driven guidelines for lipidomics implemented in a publicly available web-based questionnaire (https://lipidomicstandards.org/reporting_checklist/). Its main purpose is to describe all essential steps of lipidomic experiments in a standardized way (Fig. 1). The primary checklist output, a PDF document, is intended to assist editors and referees in reviewing research studies containing lipidomic data. In a second step, this report can be made publicly available under a Digital Object Identifier (DOI). Although PDF reports could be made available in the supplementary material of individual studies, it is preferable to include the DOI of the report in the final publication, making it available to readers and ensuring findability and accessibility (5). We note that the checklist can also be viewed as a guideline on ‘good lipidomics practice’ for both new and experienced lipidomic investigators.

Registration is required to access the interactive checklist document, allowing for archiving and future revision. Note that this account is only required for generating and maintaining checklists and is not required to have an ILS membership to register. The users are guided through the entire framework in eight steps. To maximize usability, sections and input fields have drop-down options with common answers and additional explanations, including links to references or literature. There are several options for checklist reports: 1) Update to change the report. 2) Copy to reuse the report in another study. 3) Download a PDF of the report. 4) Publish the report to get a DOI at Zenodo (once published, it is not possible to modify or delete such permanent reports). 5) Dump the report in JSON (JavaScript Object Notation) format. In addition to reusing the entire report, users can also reuse parts of the report, such as sample handling data and lipid class identification/quantification data, for another lipidomics experiment (see steps 2 and 5).

### Step 1: registration and workflow selection

**Fig. 2:**
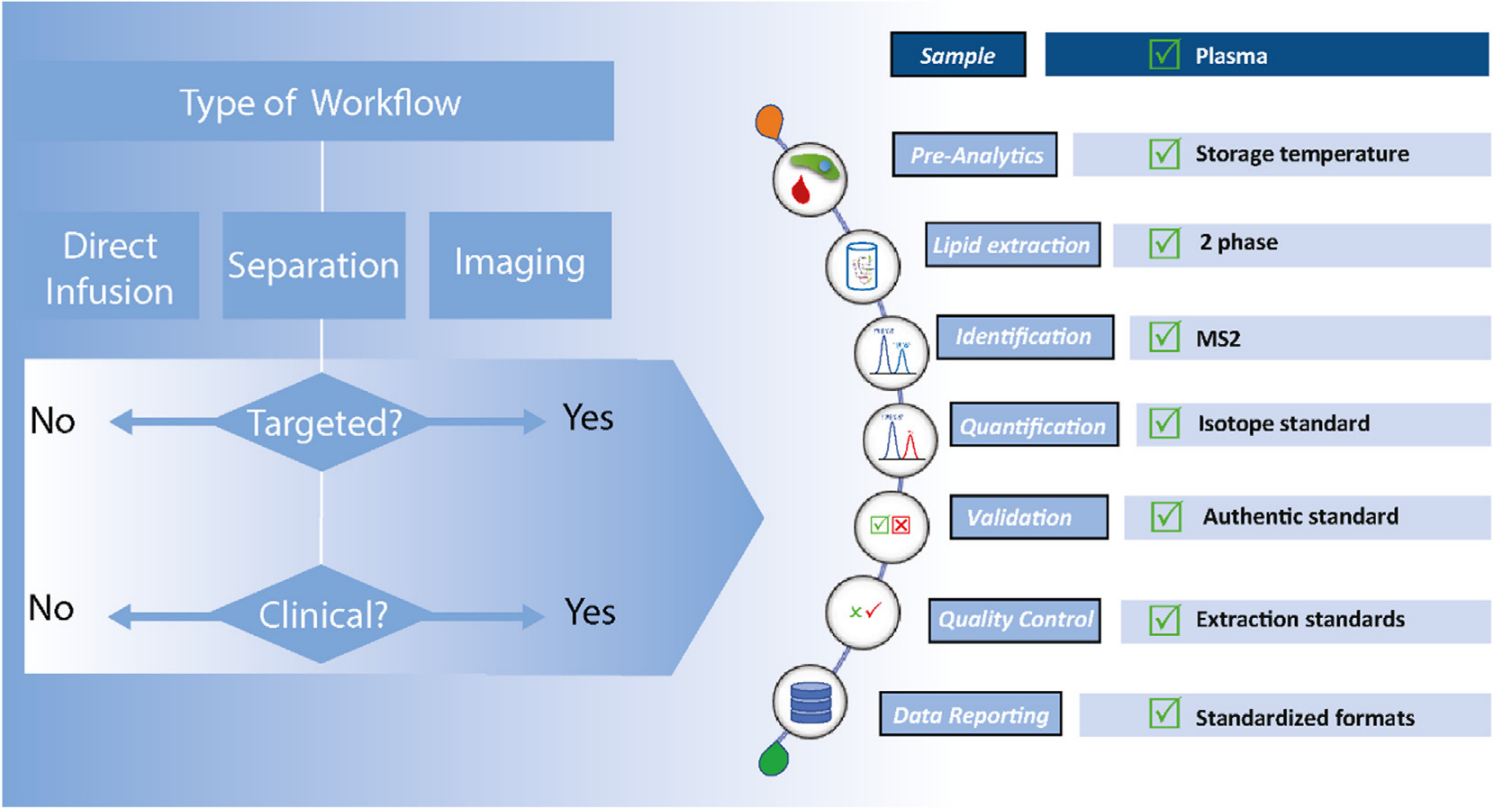
Outline of lipidomics workflow.

To generate a reporting checklist for a lipidomic study, a user first provides basic information that includes the study title, name, institution, and email address. This is followed by specifying if the analysis was targeted or untargeted, and whether it is considered clinical lipidomics (Fig. 2). These definitions are necessary to evaluate the workflow, as clinical lipidomics, for example, require more stringent criteria than screening approaches or studies in biological model systems.

Clinical lipidomics refers to studies on human samples, typically blood plasma/serum, obtained from different patient cohorts using a quantitative and validated method (7). It refers not only to studies using a clinical diagnostic method (ie, a method validated for patient diagnosis according to specific regulatory guidelines) but also to studies aimed at developing and validating lipids for clinical applications. Such lipid data are derived from lipidomic analysis but not from simple diagnostic tests such as enzymatically determined triglycerides, and LDL/HDL cholesterol.

A checklist for workflows based on imaging mass spectrometry is currently under development and will be the subject of a future report.

### Step 2: sample overview and preanalytics

In this section, the user enters the sample material (e.g. blood plasma, cells, tissue, plants, food, etc.), collection methods and storage, as well as additional steps are taken to stabilize the targeted lipids.

**Fig. 3:**
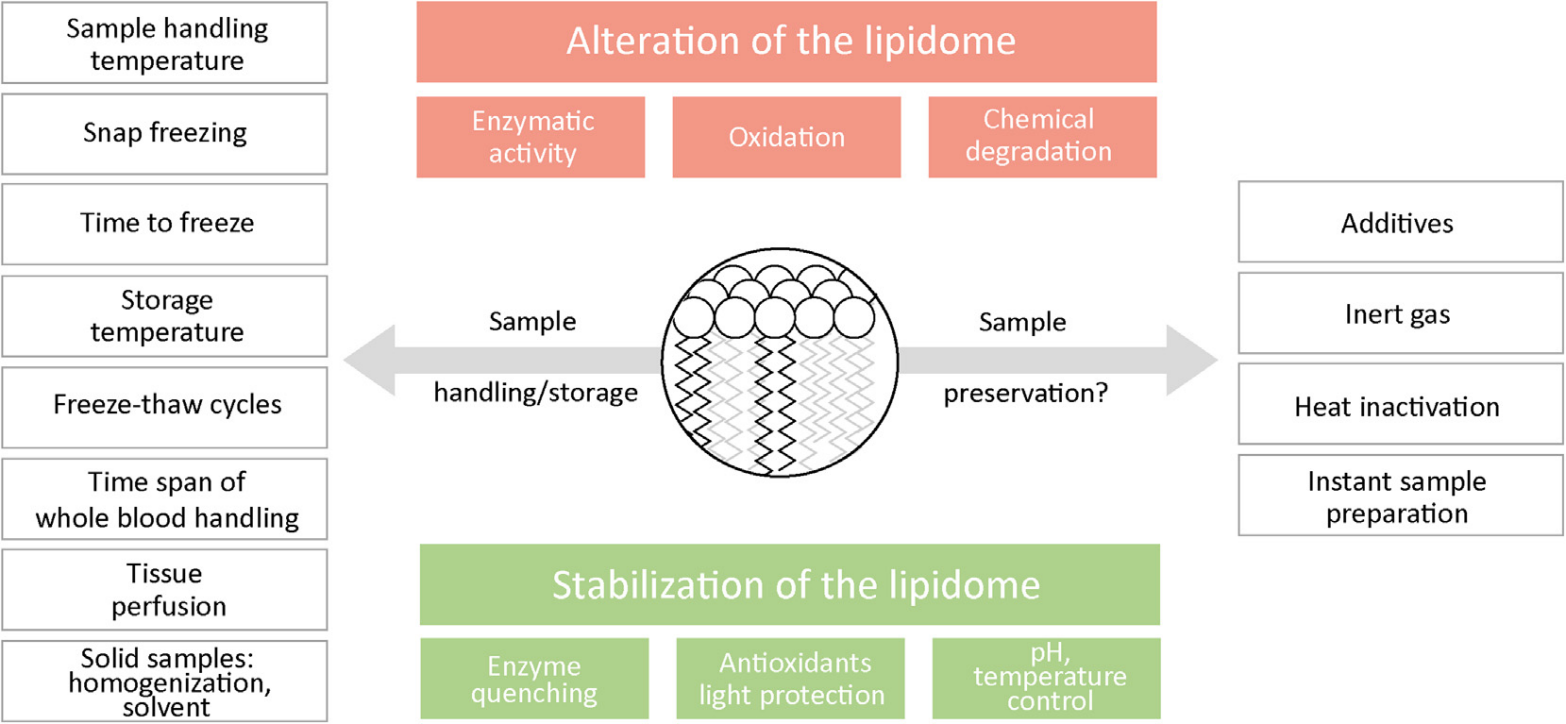
Preanalytics, sample handling and preservation.

The lipidome in biological matrices is susceptible to changes, primarily through oxidation and enzymatic activity, as well as chemical degradation (8, 9) (see Fig. 3). These processes can be mitigated through physical and/or chemical treatments. Very low temperatures (eg −80°C) are very effective in blocking such processes (8). Therefore, details regarding temperature, time for initial sample handling, storage, and freeze/thaw cycles may be provided as optional.

Additional precautions may be required for sample material with high enzymatic activities. For instance, liver homogenates containing 50% methanol still exhibit high lipolytic activity, resulting in a significant increase in ceramide and lysophospholipids (10). It has been reported that the addition of phenylmethanesulfonyl fluoride (PMSF) to plasma inhibits lipolytic activity (11). In plant material, a high phospholipase D (PLD) activity that generates phosphatidic acid (PA) can be inhibited by heat treatment in isopropanol (12). The application of heat was found to disrupt the activity of phospholipases in sample matrices of plant origin (13).

Careful sample handling is crucial when analyzing low-abundant and potentially bioactive lipids, such as lysophospholipids and oxygenated fatty acids. This is particularly important because lipid oxygenation can occur not only by enzymatic reactions but also by chemical reactions with radicals and can be induced by light and heat (14, 15). To prevent artifactual oxidation, radical scavengers such as butylated hydroxytoluene (BHT), and metal chelators (e.g. EDTA) can be added, or samples can be stored under an inert gas, typically nitrogen or argon, to reduce the presence of oxygen (8, 9, 15).

When reporting on preanalytics for certain sample types, it is important to provide additional details, eg the time required for plasma/serum separation (16), whether tissues were perfused prior to collection to remove circulating lipids, or which solvent was used to homogenize solid samples (17).

A given study may contain multiple sample sets collected and preserved under different conditions. For convenience, sample set details are stored online, accessible only by the owning user, and can be selected for the current or be reused in future workflows (see “Tips and tricks for completing the checklist” for detailed description).

### Step 3: lipid extraction

Step 3 provides details of the lipid extraction method, which is a crucial step in any lipidomic experiment (9). The primary aim of extraction is to isolate the lipids of interest from a sample while removing other macromolecules such as peptides, proteins, sugars and inorganic components, such as salts, that may interfere with analysis by MS.

**Fig. 4:**
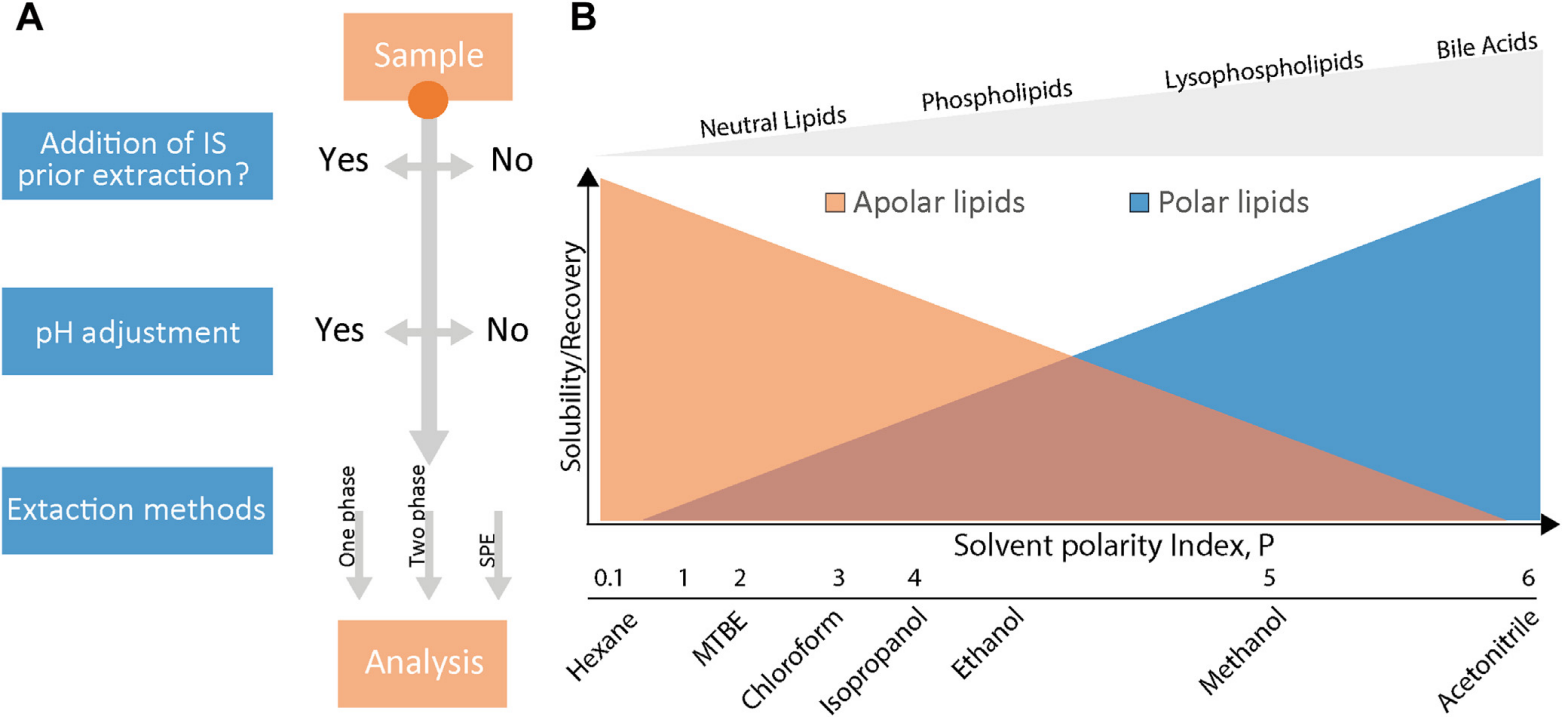
A: Methods and conditions applied for lipid extraction. B: Solubility and recovery in lipid extractions are determined by the polarity of the lipids and solvents. Solvents (polarity index) and lipid classes are ordered by increasing polarity (gray triangle).

Lipids are commonly extracted using either 1-phase or 2-phase extraction methods. In 1-phase extractions, a supernatant is separated from a precipitate by centrifugation. In 2-phase extractions, an organic (non-polar) phase is typically recovered from a polar (aqueous) phase, resulting in a purified extract. Both methods rely on the principle of 'like dissolves like', which means that nonpolar substances are more soluble in nonpolar solvents, while polar and ionic substances are more soluble in polar solvents (Fig. 4). Therefore, the careful selection of extraction solvents is important in relation to solubility. For example, non-polar lipid classes such as triglycerides and cholesteryl esters are almost completely lost during methanol precipitation due to their low solubility (18).

Solid-phase extraction, which can provide selective retention and elution of lipids from a stationary phase, can be used to isolate specific lipid classes. In the checklist, if the 'Other extraction method' option is selected, then this alternative method should be specified, ideally with a DOI referring to the extraction protocol.

Extraction and recovery of lipids from biological samples may be influenced by pH. For example, the recovery of acidic lipids in organic solvents may increase at low pH. A citric acid-buffered butanolic extraction (pH of 4) has been shown to increase the recovery of lysophosphatidic acid (19).

It is also important to note whether appropriate internal standards, (e.g., stable isotope, odd carbon acyl chain) were added to the samples prior to lipid extraction. The addition of internal lipid standards prior to extraction is required if quantitative measurements are performed as they correct for variations in recovery between samples.

Finally, details of sample derivatization, if applicable, are also collected in this section.

### Step 4: analytical platform

**Fig. 5:**
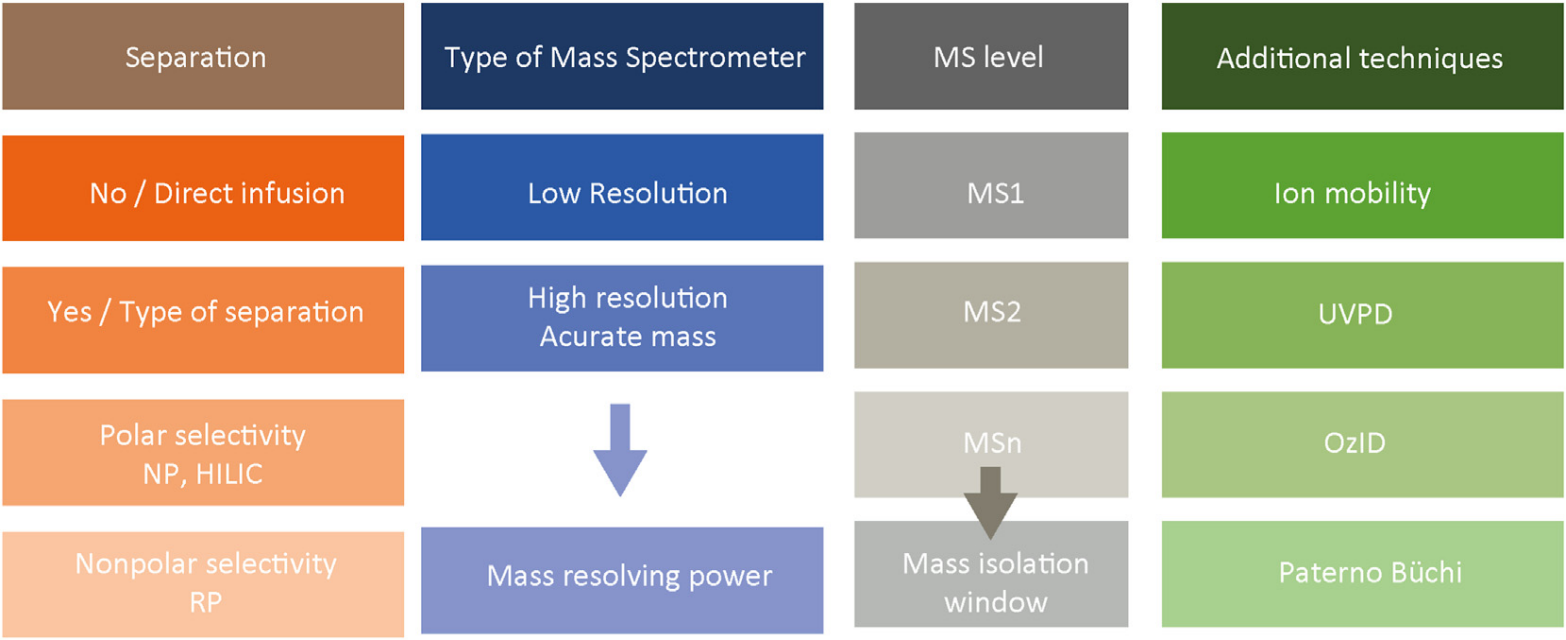
Definition of the analytical platform.

This section summarizes the analytical methods used in the respective workflow including details about separation techniques (if applied), mass spectrometric analysis, and potential additional separation techniques (Fig. 5).

**Fig. 6:**
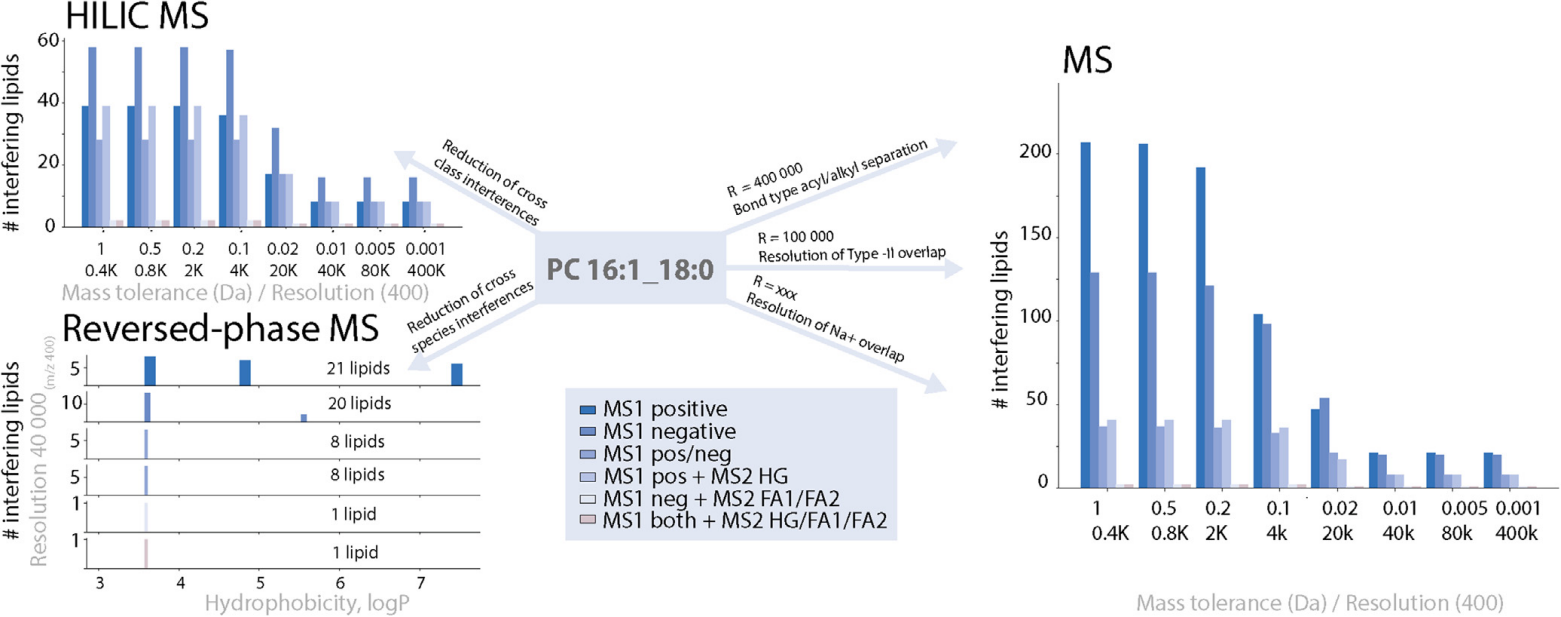
Reducing the complexity of the lipidome using mass spectrometry and separation-based analysis. For this computational experiment, all phospholipids, sphingolipids, sterols, and free fatty acids from LIPID MAPS (46) were considered. Here, PC 16:1_18:0 serves as a reference. For computing precursor masses, combinations of both lipid class common adducts and up to two ^13^C isotopes were taken into consideration. Interfering lipids are lipids that share the same set of features (mass-to-charge ratio; *m/z*) with the reference lipid within a given mass tolerance. Features are: positive/negative precursor ions, positive fragment ions *m/z* 184.07 (head group HG), negative fragment ions at *m/z* 255.23 (fatty acyl FA_1_) and *m/z* 281.24 (FA_2_). With increasing resolution and pre-separation the number of interfering lipids clearly can be reduced. Separation in the reversed phase is related to the lipophilicity or hydrophobicity of the compound displayed as a logP value.

Natural lipidomes can comprise thousands of different molecules (20), making the separation of such complex mixtures a significant challenge during analyses. The choice of analytical method can dramatically influence the number of lipids detected (Fig. 6). When separation techniques are applied, it is important to specify the type, such as gas (GC) or liquid chromatography (LC), as well as its selectivity mode (21). Polar selectivity, typically normal phase (NP) (22) or hydrophilic interaction liquid chromatography (HILIC) (23) separates lipid classes based on their polar components, such as head groups, while reversed-phase liquid chromatography (RPLC) (24) separates lipid mixtures based on their nonpolar moieties, usually their acyl chains.

**Fig. 7:**
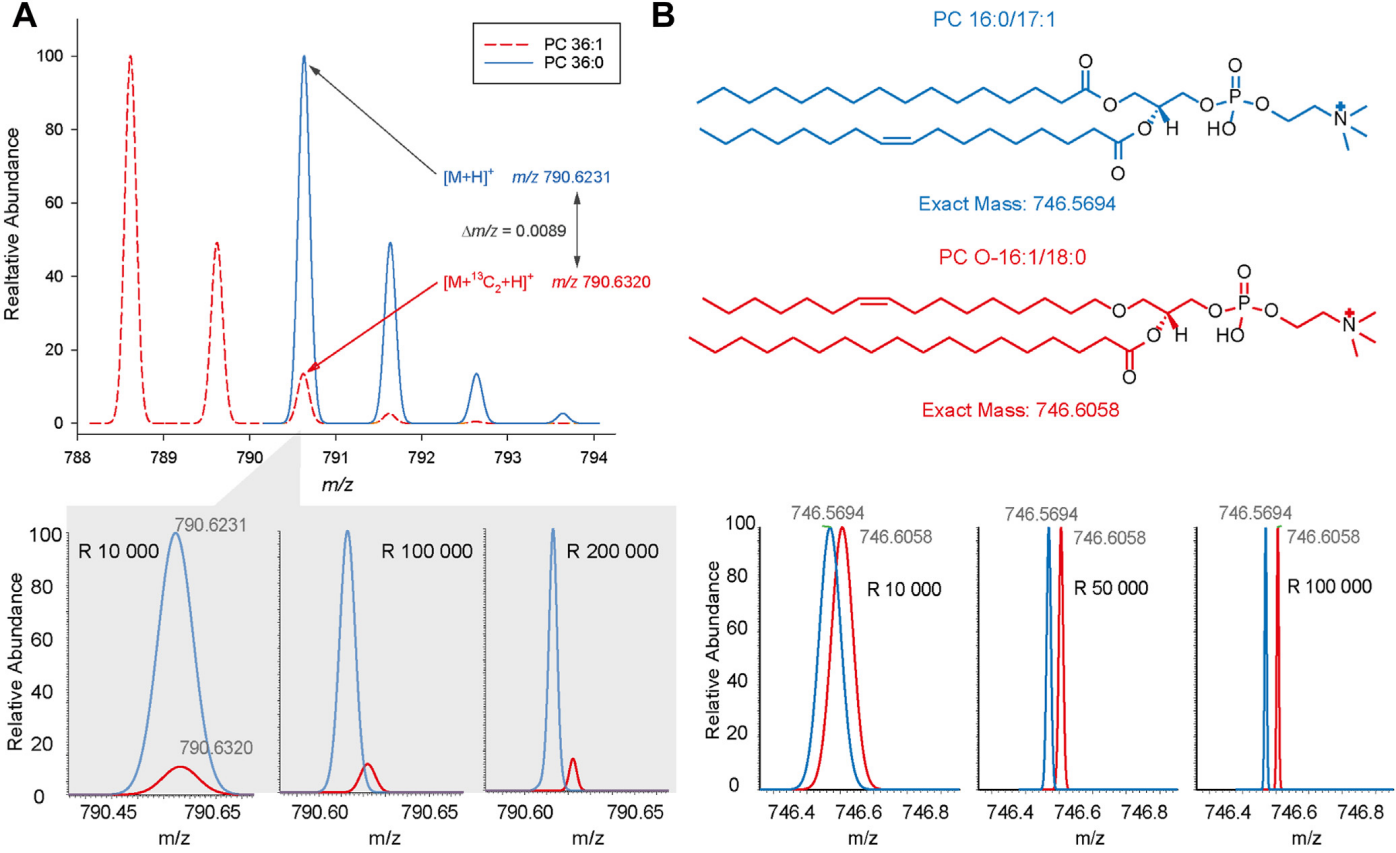
Sufficient mass resolving power (R) can separate isobaric overlaps that are commonly present in lipidomic analysis, such as (A) Type-II overlaps occurring for double bond series and (B) isobars resulting from different bond types as seen for diacyl PC 16:0/17:1 and alkyl/acyl PC O-16:1/18:0.

Most lipidomic workflows utilize mass spectrometry for analysis. Therefore, the checklist includes information on instrument type and manufacturer, ion source, level of analysis (i.e., MS^1^, MS^2^, and MS^3^), and the applied resolving power of the mass spectrometer (usually specified in the MS method for mass *m/z* 200 or 400). The latter can be used to distinguish isobaric species, which are ions with the same nominal mass but not exact elemental composition. Examples of isobaric overlaps (see also https://lipidomicstandards.org) are given in Fig. 7. In lipidomics, important isobaric interferences include the Type-II isotopic overlap (mass difference of 9 mDa) occurring in double bond series within the same lipid class, and the ambiguity resulting from different bond types in lipids with a glycerol-backbone (mass difference of 36 mDa) (Fig. 7). Notably, the mass resolution is not only dependent on the type of analyzer but can also be related to *m/z*. While the resolving power of Orbitrap analyzers decreases with the square root of *m/z*, it remains constant for time-of-flight (TOF) analyzers. Thus, Type-II overlap may be resolved for lysophospholipids but not for triglycerides in a regular medium-resolution Orbitrap spectrum, and the ratio of overlapping isobars may also affect their peak resolution (25).

Isomeric ions that have the same elemental composition cannot be distinguished by their *m/z* values. Therefore, tandem mass spectrometry (MS^2^) is often used to differentiate between lipid classes and/or acyl chains by their respective product ions. When using MS^2^ or MS^3^, it is important to specify the width of the isolation window. A wider isolation window (eg, greater than ±0.5 *m/z*) substantially increases the number of co-isolated ions. Mass spectrometer manufacturers typically recommend using wider isolation windows to increase sensitivity at the risk of reduced analytical specificity.

**Fig. 8:**
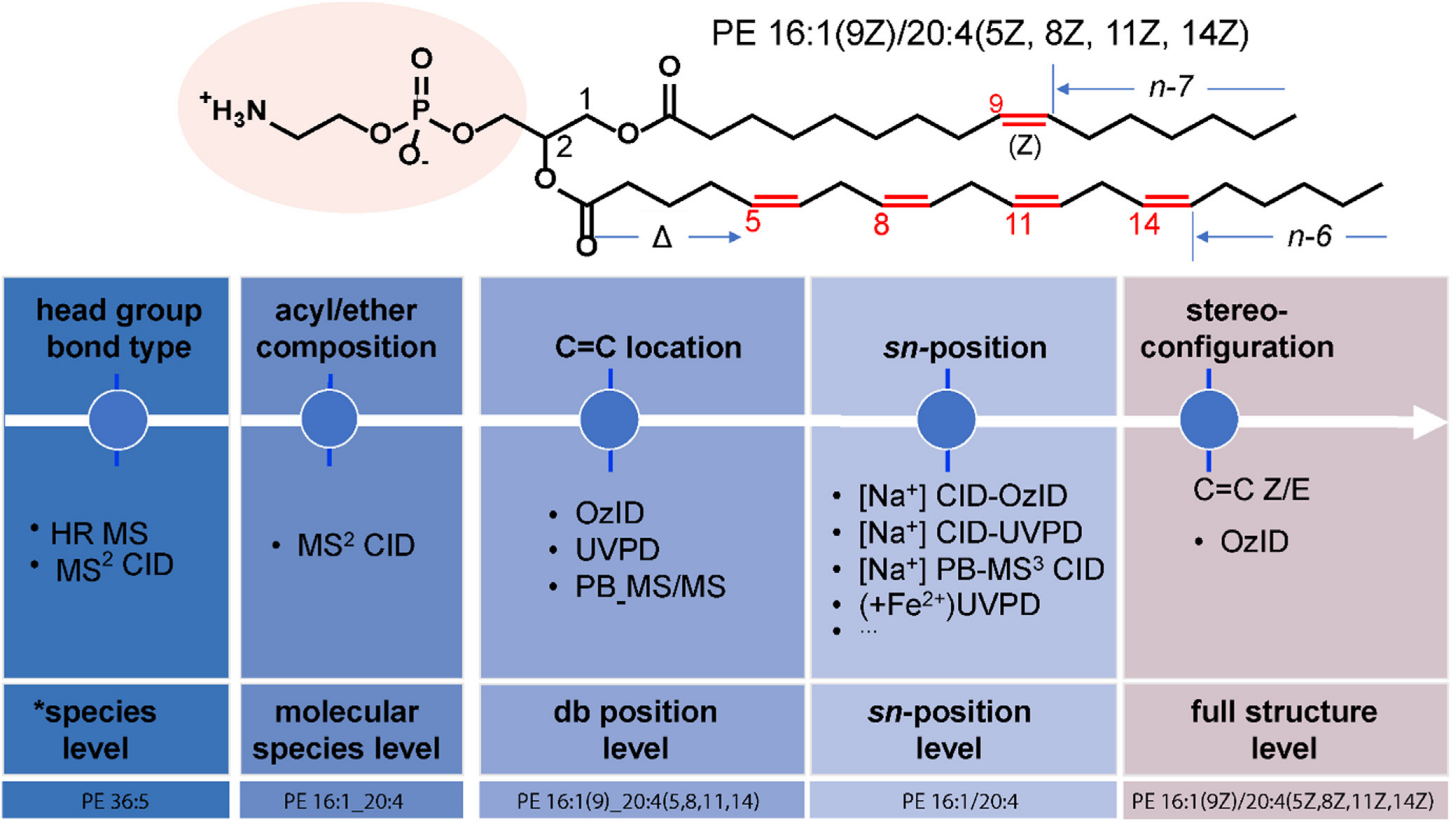
Elucidation of lipid molecule structure and related annotation/structure level. Source: Adapted from Zhang *et al.* (31).

Additional analytical dimensions, such as ion mobility spectrometry (IMS), gas phase reactions like ozone-induced dissociation (OzID) (26) and ultraviolet photodissociation (UVPD) (27), or derivatization reactions like Paternò-Büchi (PB) (28), can enhance the structural resolution of the workflow (Fig. 8). Details on these additional dimensions are collected in the following section.

For convenience, identification and quantification details are stored online and can be selected or copied (see “Tips and tricks for completing the checklist” for a detailed description).

### Step 5: lipid identification and quantification

This section aggregates all information on how the reported lipids were identified and quantified. Annotation of the lipids is based on the latest version of the shorthand notation for MS-derived lipid structures (3) (for updates, see also https://www.lipidmaps.org/shorthand_nomenclature), which includes hierarchical architecture to reflect only structural details provided by mass spectrometric analysis (Fig. 8). The information in this section is crucial to examine the fidelity of lipid identification and quantification accuracy of the reported data.

A key principle of lipid identification is that the annotation of the lipid should provide only those structural details that are supported by the analytical method. For example, high-resolution MS^1^ with accurate mass or detection of a lipid class-specific fragment in MS^2^ only allows annotation at the lipid species level (i.e., the sum of the composition of the variable components). Annotation at the molecular lipid species level requires additional information on the variable components, typically the acyl chains. It is recommended to report such fragment ions according to the nomenclature proposed by Pauling *et al.* (29). Fragment ions are suggested for common lipid classes as implemented in LipidCreator (30) and ALEX123 (29).

To specify additional structural details such as *sn*-positions or double bond locations by mass spectrometric analysis, additional analytical dimensions or chemical derivatization need to be used (31). When applying chromatographic separations, it is important to indicate whether an algorithm has been used to predict or verify the separation, such as retention time prediction models (32). There are several important quality assurance and quality control (QA/QC) steps to minimize misidentifications: 1) Check for overlaps with isobaric molecules (https://lipidomicstandards.org/isobaric-overlap/) for example in double bond series (Type-II, Fig. 6), other lipid classes ([PI 34:1-H]^–^ and [PS 40:6-H+^13^C]^–^ Δ*m/z* = 0.002) or different ions ([PC 34:1+Na]^+^ and [PC 36:4+H]^+^ Δ*m/z* = 0.002). 2) Check for overlaps with isomeric molecules (https://lipidomicstandards.org/isomeric-overlap/) such as phosphatidylcholine (PC) and phosphatidylethanolamine (PE) (*m/z* [PC 32:0+H]^+^ = *m/z* [PE 35:0+H]^+^). 3) Check whether the precursor ion could result from in-source fragmentation (https://lipidomicstandards.org/in-source-fragmentation/) such as loss of water (*m/z* [Cer 34:1;O2+H]^+^ = *m/z* [Cer 34:0;O3+H-H_2_O]^+^. 4) Use of a standard to verify ionization (which adduct ions are formed), if applicable, which fragment ions or retention time are observed for the respective lipid class at the selected analytical conditions. 5) Demonstrating the lipid being reported is not observed in the background (see also the definition of the blank samples in step 6). 6) Ensuring the signal is above the limit of detection (LOD), typically defined as a signal-to-noise (S/N) ratio greater than three). Determination of S/N must be performed without smoothing and needs to include a sufficient number of data points (33).

**Fig. 9:**
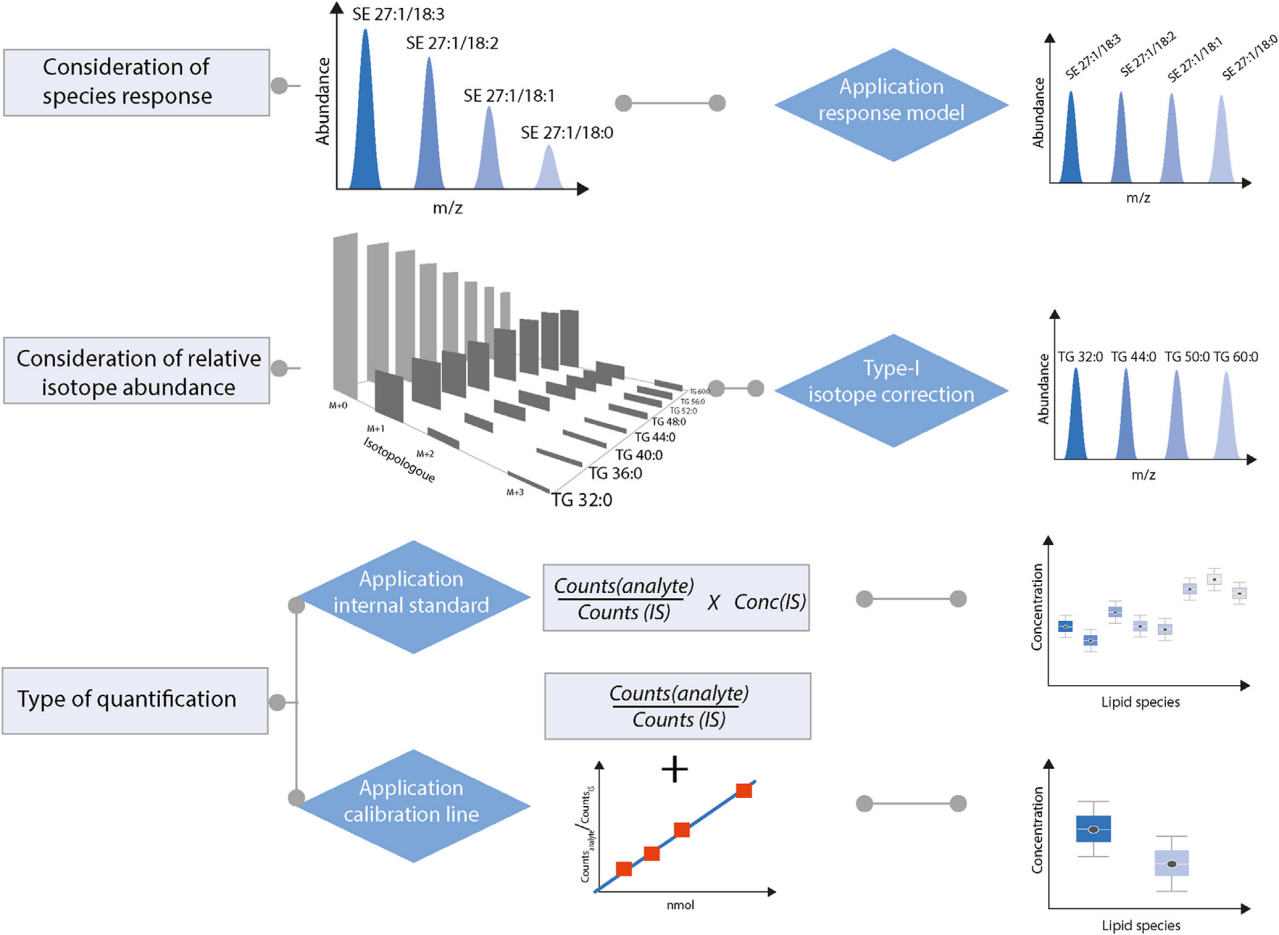
Lipid molecule quantification.

Mass spectrometric quantification requires the application of internal standards (IS) due to the substantial influence of the sample matrix on the ionization process, particularly in electrospray ionization (ESI). It is highly recommended to use at least one IS per lipid class (4) since the ionization efficiency of a lipid molecule in ESI is strongly correlated to its polarity, typically its (polar) head group characteristic for the respective lipid class. In addition to reporting details on the IS, it is important to specify the type of quantification (Fig. 8). This includes whether the concentration was calculated based solely on the amount of the IS used (single point calibration) or if calibration curves were utilized. If calibration curves were used, it is important to note whether they were generated in a solvent or sample matrix and which lipids were included in the calibration. For certain lipid classes, the analytical response is also influenced by structural characteristics, such as the length of the acyl chain and the number of double bonds. In these cases, accurate quantification may necessitate the use of response models, as demonstrated for cholesteryl esters (34), and ceramides (35). The analytical response is typically more uniform when MS^1^ is applied. When MS^2^ is used, it also depends on which fragments are used for quantification. For example, PC species analyzed using the lipid class-specific fragment *m/z* 184.0733 show a similar response (36), whereas accurate quantification using only carboxylic acid fragments requires a response model (37). Another factor that affects the analytical response is the Type-I isotopic effect, which refers to the decrease in the proportion of monoisotopic peaks as the number of carbon atoms increases (Fig. 9). This effect can be corrected by adjusting the intensities of the monoisotopic peaks based on the theoretical isotopic pattern. Finally, concentrations must incorporate the volume for liquid samples such as plasma, the protein or DNA content for cultured cells, or wet weight for solid material such as organs.

Every lipidomic workflow relies on software to process the raw data, including isotope correction to minimize, to identify, and quantify lipid molecules. Therefore, information concerning the applied software (including databases and libraries) is collected which includes steps for data transformations such as smoothing, centroiding, lock mass correction or batch corrections. In case, multiple software tools are, the user can specify this in a free-text field.

### Step 6: quality control (QC)

This section of the checklist collects information regarding QC aspects of the lipidomic experiment.

**Fig. 10:**
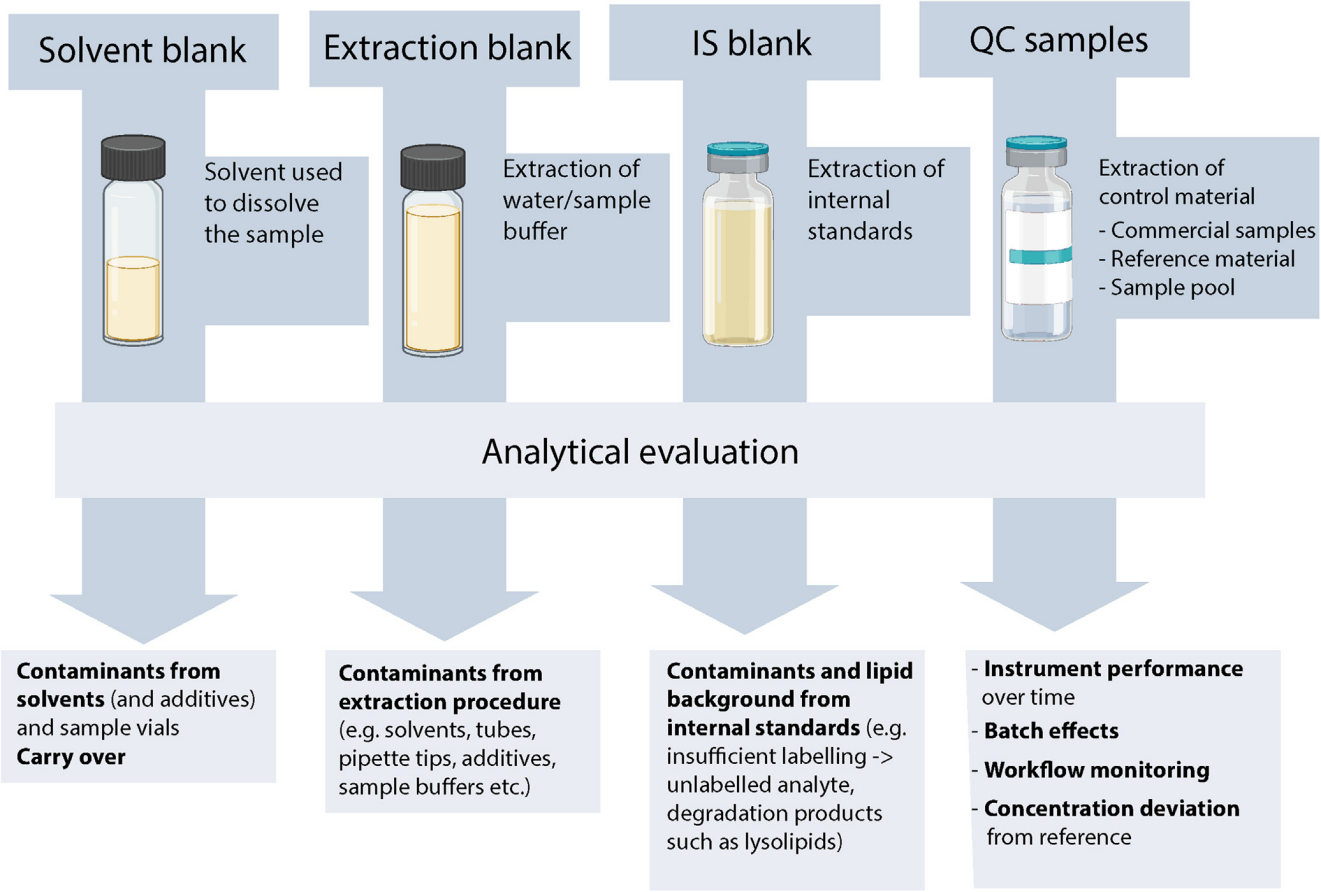
Blank and QC samples used for analytical evaluation of the workflow. Blanks provide insight into background lipids/contaminants and their origin. Quality Control (QC) samples are used to evaluate the reliability of the analysis.

Blank samples are essential for identifying interferences, especially lipids that may not originate from the sample material or chemical contaminants that can be misidentified as lipids. For instance, solvents, additives for ionization, vials, extraction tubes, pipette tips, etc., may contain such interferences (38). These contaminants can be monitored by different blank samples, including the solvent, extraction, and internal standard blanks (Fig. 10). Solvent and extraction blanks are used to check for contaminants resulting from solvents, chemicals, tubes, and plastic pipette tips. Solvent blanks are also suitable for monitoring sample carryover. The internal standard blank is necessary to identify interferences, especially lipid background that may arise from the internal standards. For instance, insufficient labeling of stable isotope-labeled standards may lead to unlabeled lipid molecules while degradation of complex lipids may result in an artificial increase in lysolipids. When interferences are present, affected lipids can either be excluded from analysis or subtracted if they represent a stable background.

QC samples typically have the same matrix as the samples being analyzed, often pooled samples. QCs are valuable for monitoring instrument performance and batch-to-batch variation. They are treated in the same way as the regular samples and, therefore, allow control of the entire lipid analysis workflow (39). QC monitoring is mandatory for quantitative lipidomics workflows and typically includes multiple QCs. For clinical lipidomics, QCs may include acceptance ranges for the respective lipid concentrations. When commercial or reference samples are included, concentrations can be related to reference values and used for alignment with other laboratories with different workflows.

### Step 7: method validation

**Fig. 11:**
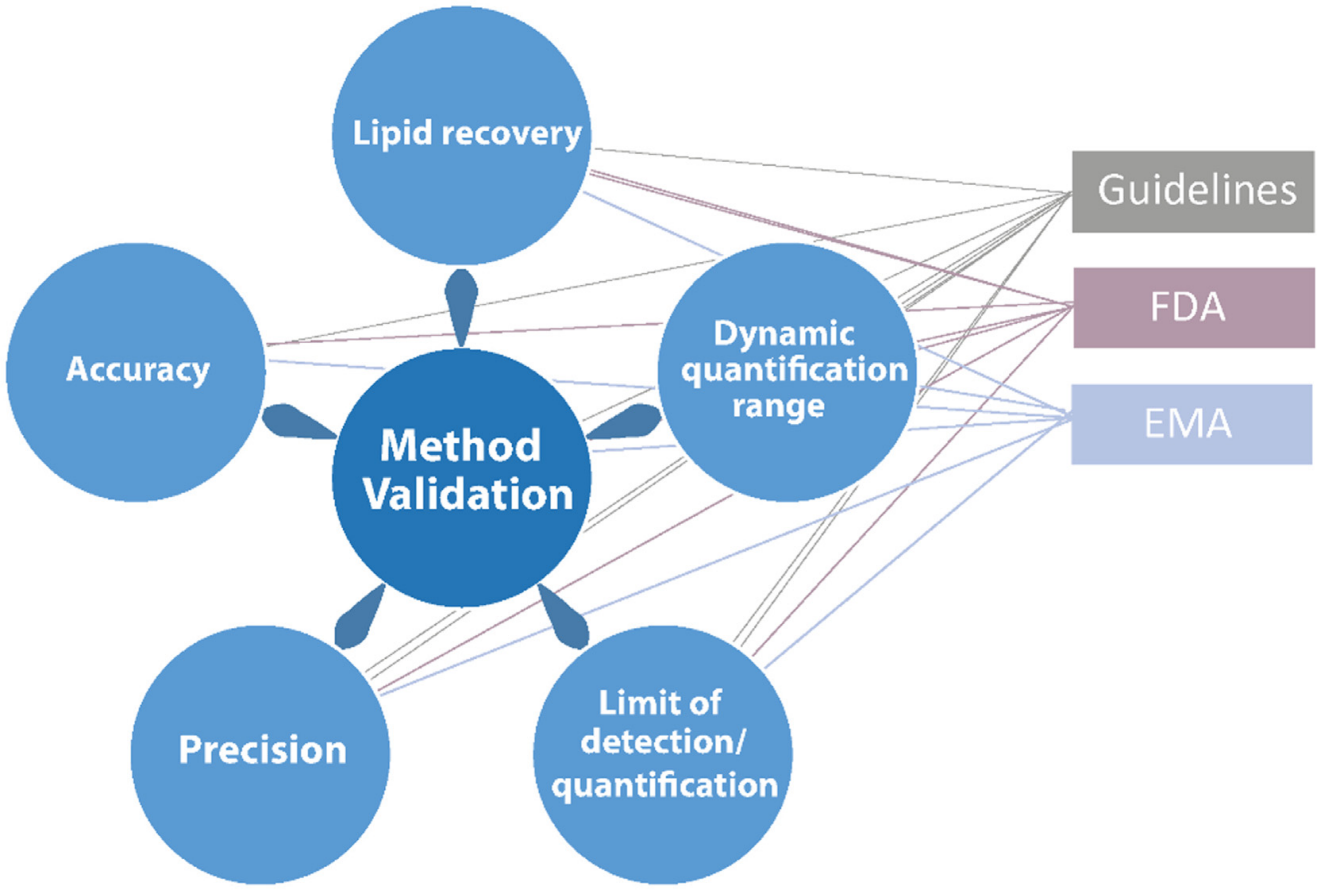
Parameters that are typically evaluated in method validations and related guidelines.

This section seeks information regarding the validation of the reported lipidomics workflow. Basic method characteristics that are typically evaluated during method validation include the recovery of lipids, dynamic linear range, the LOD, and limit of quantification (LOQ) as well as precision and accuracy (Fig. 11). Determining these basic characteristics is usually sufficient for quantitative research assays. However, clinical assays require additional validation, which often involves following guidelines for bioanalytical method validation provided by the European Medicines Agency (EMA) (https://www.ema.europa.eu/en/ich-m10-bioanalytical-method-validation-scientific-guideline) or the U.S. Department of Health and Human Services Food and Drug Administration (FDA) (https://www.fda.gov/regulatory-information/search-fda-guidance-documents/m10-bioanalytical-method-validation-and-study-sample-analysis). These guidelines provide recommendations for chromatography-based methods, covering not only the determination of basic characteristics but also additional parameters such as selectivity/specificity, matrix effect, carryover, dilution integrity, and analyte stability.

### Step 8: reporting summary

**Fig. 12:**
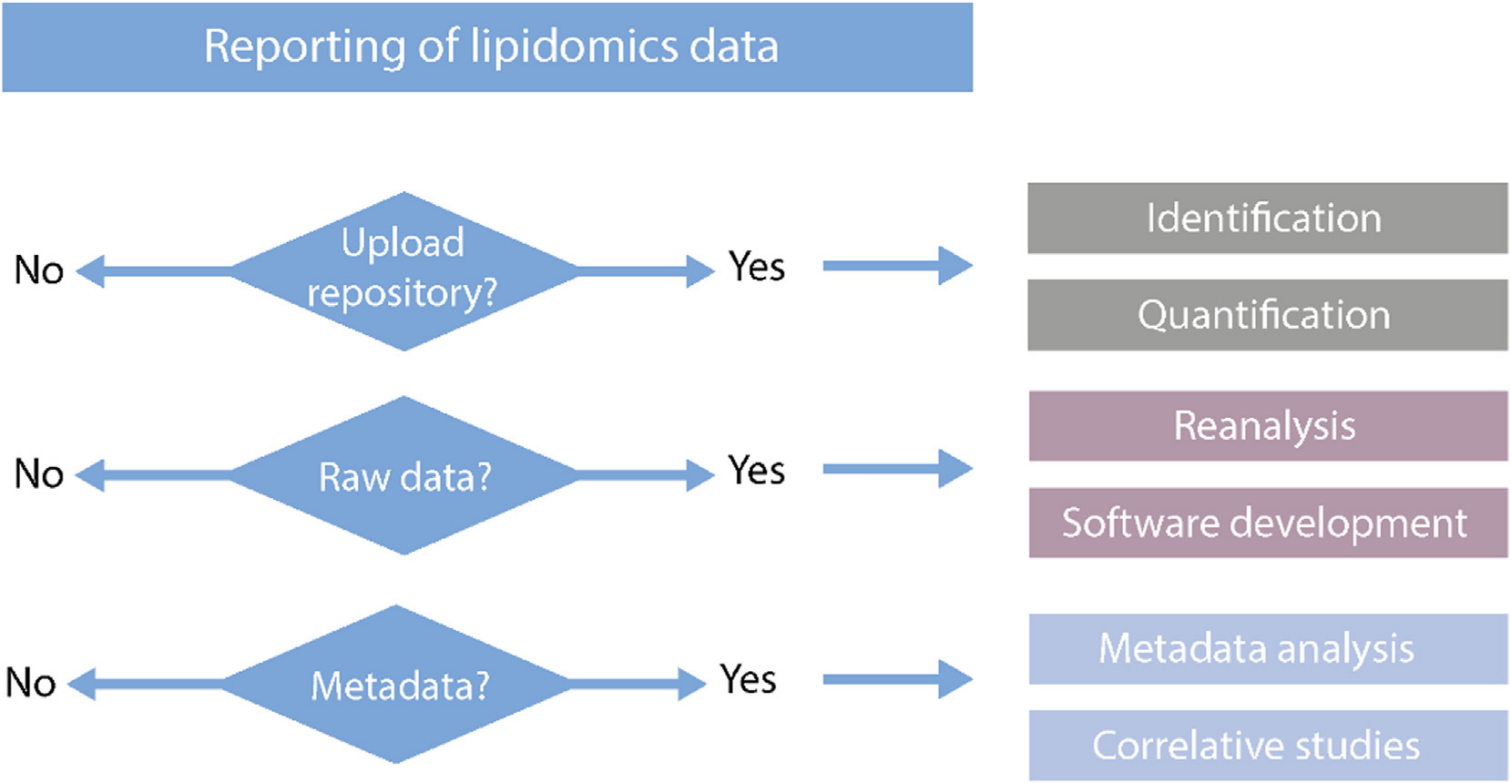
Reporting of data.

In the final section of the checklist, the user is asked whether a public repository is used to store the experimental data, such as raw and result files, containing results of identification and/or quantification (Fig. 12). A simple provision of all raw data in a spreadsheet file on online storage may satisfy the requirement of publishing the data. However, without providing metadata such as names (identifiers) of measured samples, or result files, a first assessment might be challenging when determining if the data can be evaluated for other purposes. Repositories such as MetaboLights (40) and Metabolomics Workbench (41) fit these requirements, as well as Panorama, which is established in the proteomics field (42). These databases should provide both the information type and the actual information, eg, ‘lipid identifier’ → PC 16:0_18:1. The provision of the complete record is also standardized, usually with open formats, for instance, in.mzTab-M (43) format and/or standard spreadsheet formats. Additionally, the user can specify a list of raw and processed file names and link the checklist to the data repository. In cases where data cannot be disclosed in a public repository, eg clinical and population datasets, the user should enter a link to the resource that allows for mediated access to the data or provide contact details of the study authors or stewards. This information can be, e.g., written in the additional comments field in this section.

### Tips and tricks for completing the checklist

**Fig. 13:**
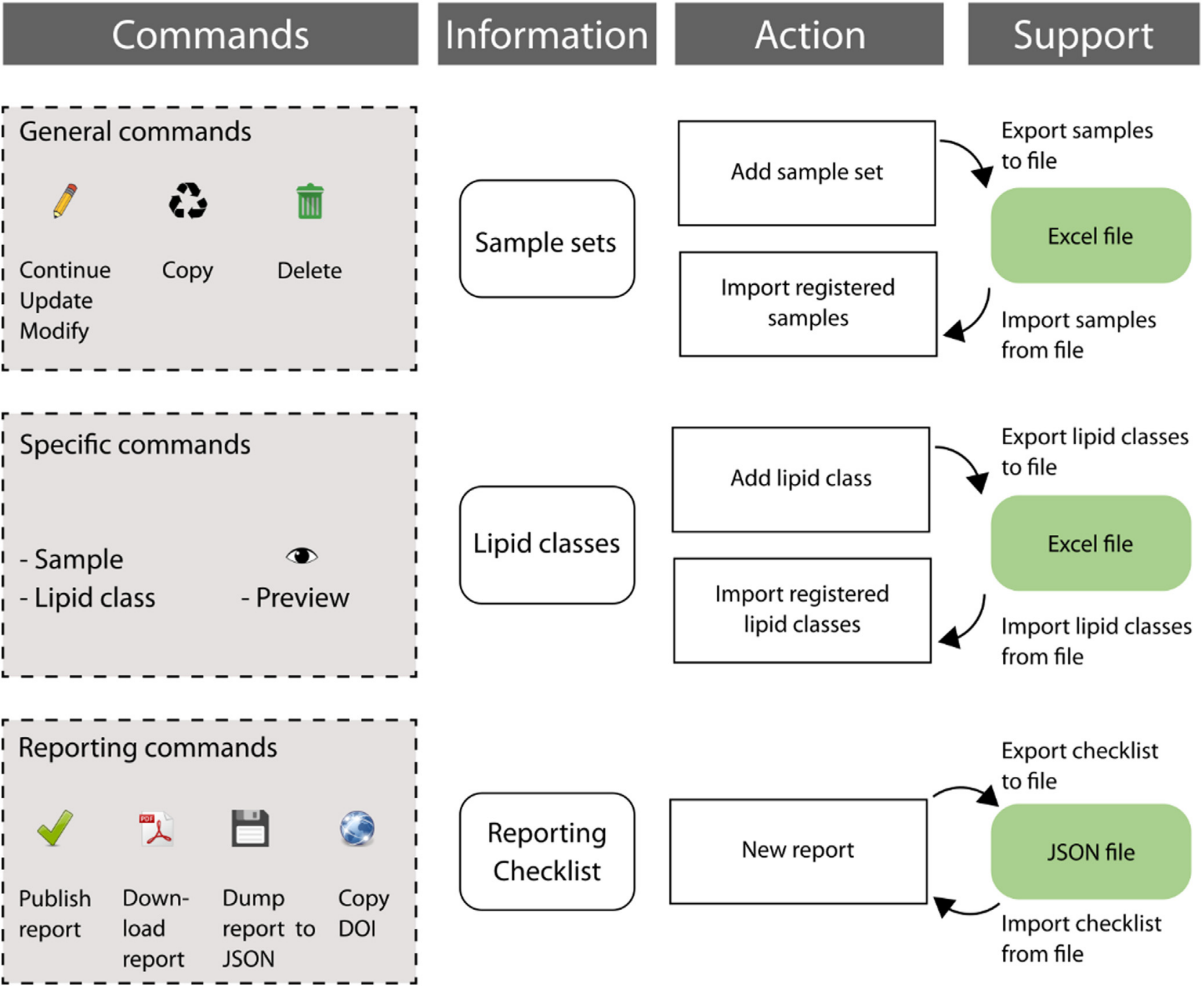
Reporting checklist data handling.

To provide an overview of the details required to complete a checklist and to facilitate its generation, sample PDFs of the checklist are provided as supplementary data and on the home page. They have also been published on Zenodo (https://doi.org/10.5281/zenodo.13692575, https://doi.org/10.5281/zenodo.13692579, https://doi.org/10.5281/zenodo.13692581). The generation of the first checklist may take some time, depending on the number of sample sets and lipid classes included in the study. However, once a checklist exists for a particular workflow, it can be copied and modified for the particular study, typically by addition/modification of sample set and lipid class identification/quantification details, facilitating a faster checklist generation (Fig. 13).

As one lipidomic workflow may cover numerous lipid classes, reporting the required identification/quantification details could be tedious. Therefore, the checklist provides several options to save time: 1) Lipid class details can be imported from previous reports. 2) Lipid class details can be exported to and imported from Excel spreadsheets. 3) Lipid class details can be modified/previewed/deleted.

This allows the user to enter details for just one lipid class and then copy and modify that entry. For example, for glycerophospholipids, it may be necessary to only change the lipid class-specific fragment ion and internal standard. Such details could be maintained in an Excel spreadsheet and quickly modified by copy/paste actions and then uploaded to the respective report. Similarly, sample set details can be uploaded and reused to save time.

In collaborative projects, it may be necessary to collect checklist information from different working groups. Typically, sample details are provided by biologists and physicians, and analytical method details are provided by the lipidomics laboratory. To facilitate the completion of the checklist in such projects, we aim to introduce a shared checklist in the future. For now, we recommend using a shared account to complete the details. Of note, when lipidomic data are provided by commercial vendors or core facilities, it is recommended that the data providers be made aware that method details should also be filled into the checklist.

## Discussion

The Lipidomics Minimal Reporting Checklist provides an automated easy-to-use pipeline that facilitates the reporting of all crucial steps of lipidomics workflows in a single standardized document. The checklist not only assists users in determining what should be reported but also provides essential information on the reasons behind certain procedures, such as using internal standards, naming, or applying isotope correction in lipidomics experiments. In this article, we provide multiple educational examples to highlight the necessity of such reporting to improve understanding of the complexity of lipidomics and the implications this complexity has on quality data output.

Importantly, the checklist is a community-driven effort and thrives on community engagement. We actively encourage users to provide feedback and submit their requests, as this affords continuous improvements of the lipidomics checklist. Currently, more than 400 reports have been generated. In the future, we plan to tighten the link to repositories storing the qualitative and quantitative information to provide a platform for intuitive data interoperability and re-usability within the community. Using this valuable insight will foster the advancement of lipidomics and streamline decision-making processes, enabling researchers to make informed choices more efficiently.

In addition to guiding researchers in lipidomics workflows, the checklist also assists editors and reviewers in reviewing lipidomic studies. This can contribute to improving the quality of data in lipidomics research. Furthermore, the checklist output, a PDF document, can be made immutably publicly available with a DOI in Zenodo which should be provided within a manuscript submission. In addition, it is easy to link the DOI to lipidomic data even in existing data repositories (40, 41). In the future, data repositories may consider more detailed and automated quality checks that link experimental data and checklist information to a quality score. This can help readers evaluate the reported lipidomic data more effectively and to make lipidomic data FAIRer to improve reusability and reproducibility. Overall, the anticipated improvement in the quality and usability of lipidomic data will then ultimately lead to a better understanding of the complex roles of lipids in biological systems. Furthermore, the checklist framework follows harmonization efforts in other fields such as in proteomics (Proteomics Standards Initiative, https://www.psidev.info/) (44) and genomics (The Genomic Standards Consortium, https://www.gensc.org/) (45) which are important for multiomics data integration.

In conclusion, the Lipidomics Minimal Reporting Checklist is a community-based effort promoting standardization and harmonization. Currently, the ILS is working to increase awareness of the Lipidomics Checklist. On the one hand, the checklist will be presented at conferences, and on the other hand, attempts will be made to convince journals of its benefits. A future goal for the Lipidomics Minimal Reporting Checklist is to become a standard practice where everyone routinely includes the report with their publications and disseminated data.

## Data availability

There are no data associated with this report.

## Supplemental data

This article contains supplemental data.

## Conflict of interests

Kaddurah-Daouk is an inventor on key patents in the field of metabolomics and hold equity in Metabolon, a biotech company in North Carolina. In addition, she holds patents licensed to Chymia LLC and PsyProtix with royalties and ownership. Kim Ekroos is the owner of Lipidomics Consulting Ltd.

## Supplementary Data

Liver FIA-QQQ.pdf

Fibros HILIC-QQQ.pdf

Plasma nano-infusion-FTMS.pdf

## References

[1] Liebisch G., Ahrends R., Arita M., Arita M., Bowden J.A., Ejsing C.S. (2019). Lipidomics needs more standardization. Nat. Metab..

[2] Köfeler H.C., Eichmann T.O., Ahrends R., Bowden J.A., Danne-Rasche N., Dennis E.A. (2021). Quality control requirements for the correct annotation of lipidomics data. Nat. Commun..

[3] Liebisch G., Fahy E., Aoki J., Dennis E.A., Durand T., Ejsing C.S. (2020). Update on LIPID MAPS classification, nomenclature, and shorthand notation for MS-derived lipid structures. J. Lipid Res..

[4] Kofeler H.C., Ahrends R., Baker E.S., Ekroos K., Han X., Hoffmann N. (2021). Recommendations for good practice in MS-based lipidomics. J. Lipid Res..

[5] Wilkinson M.D., Dumontier M., Aalbersberg I.J., Appleton G., Axton M., Baak A. (2016). The FAIR Guiding Principles for scientific data management and stewardship. Sci. Data.

[6] McDonald J.G., Ejsing C.S., Kopczynski D., Holcapek M., Aoki J., Arita M. (2022). Introducing the lipidomics minimal reporting checklist. Nat. Metab..

[7] Burla B., Arita M., Arita M., Bendt A.K., Cazenave-Gassiot A., Dennis E.A. (2018). MS-based lipidomics of human blood plasma: a community-initiated position paper to develop accepted guidelines. J. Lipid Res..

[8] Ulmer C.Z., Koelmel J.P., Jones C.M., Garrett T.J., Aristizabal-Henao J.J., Vesper H.W. (2021). A review of efforts to improve lipid stability during sample preparation and standardization efforts to ensure accuracy in the reporting of lipid measurements. Lipids.

[9] Vale G., McDonald J.G. (2023). Mass Spectrometry for Lipidomics: Methods and Applications.

[10] Krautbauer S., Blazquez R., Liebisch G., Höring M., Neubert P., Pukrop T. (2021). Application of lipid class ratios for sample stability monitoring-evaluation of murine tissue homogenates and SDS as a stabilizer. Metabolites.

[11] Wang X., Gu X., Song H., Song Q., Gao X., Lu Y. (2015). Phenylmethanesulfonyl fluoride pretreatment stabilizes plasma lipidome in lipidomic and metabolomic analysis. Anal. Chim. Acta.

[12] Shiva S., Enninful R., Roth M.R., Tamura P., Jagadish K., Welti R. (2018). An efficient modified method for plant leaf lipid extraction results in improved recovery of phosphatidic acid. Plant Methods.

[13] Koelmel J.P., Jones C.M., Ulmer C.Z., Garrett T.J., Yost R.A., Schock T.B. (2018). Examining heat treatment for stabilization of the lipidome. Bioanalysis.

[14] O’Donnell V.B., Milne G.L., Nogueira M.S., Giera M., Helge Schebb N. (2023). Mass Spectrometry for Lipidomics: Methods and Applications.

[15] Griffiths W.J., Crick P.J., Wang Y. (2013). Methods for oxysterol analysis: past, present and future. Biochem. Pharmacol..

[16] Wang Q., Hoene M., Hu C., Fritsche L., Ahrends R., Liebisch G. (2023). Ex vivo instability of lipids in whole blood: preanalytical recommendations for clinical lipidomics studies. J. Lipid Res..

[17] Höring M., Krautbauer S., Hiltl L., Babl V., Sigruener A., Burkhardt R. (2021). Accurate lipid quantification of tissue homogenates requires suitable sample concentration, solvent composition, and homogenization procedure-A case study in murine liver. Metabolites.

[18] Horing M., Stieglmeier C., Schnabel K., Hallmark T., Ekroos K., Burkhardt R. (2022). Benchmarking one-phase lipid extractions for plasma lipidomics. Anal. Chem..

[19] Baker D.L., Umstot E.S., Desiderio D.M., Tigyi G.J. (2000). Quantitative analysis of lysophosphatidic acid in human blood fractions. Ann.N.Y.Acad.Sci..

[20] Shevchenko A., Simons K. (2010). Lipidomics: coming to grips with lipid diversity. Nat.Rev.Mol.Cell Biol..

[21] Holčapek M., Peterka O., Chocholoušková M., Wolrab D., Holčapek M., Ekroos K. (2023). Mass Spectrometry for Lipidomics: Methods and Applications.

[22] Sokol E., Almeida R., Hannibal-Bach H.K., Kotowska D., Vogt J., Baumgart J. (2013). Profiling of lipid species by normal-phase liquid chromatography, nanoelectrospray ionization, and ion trap-orbitrap mass spectrometry. Anal. Biochem..

[23] Peterka O., Maccelli A., Jirásko R., Vaňková Z., Idkowiak J., Hrstka R. (2024). HILIC/MS quantitation of low-abundant phospholipids and sphingolipids in human plasma and serum: dysregulation in pancreatic cancer. Analytica Chim. Acta.

[24] Ovčačíková M., Lísa M., Cífková E., Holčapek M. (2016). Retention behavior of lipids in reversed-phase ultrahigh-performance liquid chromatography–electrospray ionization mass spectrometry. J. Chromatogr. A..

[25] Höring M., Ejsing C.S., Krautbauer S., Ertl V.M., Burkhardt R., Liebisch G. (2021). Accurate quantification of lipid species affected by isobaric overlap in Fourier-transform mass spectrometry. J. Lipid Res..

[26] Michael J.A., Young R.S.E., Balez R., Jekimovs L.J., Marshall D.L., Poad B.L.J. (2024). Deep characterisation of the sn-isomer lipidome using high-throughput data-independent acquisition and ozone-induced dissociation. Angew. Chem. Int. Ed. Engl..

[27] Klein D.R., Brodbelt J.S. (2017). Structural characterization of phosphatidylcholines using 193 nm ultraviolet photodissociation mass spectrometry. Anal. Chem..

[28] Shi H., Tan Z., Guo X., Ren H., Wang S., Xia Y. (2023). Visible-light paternò-büchi reaction for lipidomic profiling at detailed structure levels. Anal. Chem..

[29] Pauling J.K., Hermansson M., Hartler J., Christiansen K., Gallego S.F., Peng B. (2017). Proposal for a common nomenclature for fragment ions in mass spectra of lipids. PLoS One.

[30] Peng B., Kopczynski D., Pratt B.S., Ejsing C.S., Burla B., Hermansson M. (2020). LipidCreator workbench to probe the lipidomic landscape. Nat. Commun..

[31] Zhang W., Jian R., Zhao J., Liu Y., Xia Y. (2022). Deep-lipidotyping by mass spectrometry: recent technical advances and applications. J. Lipid Res..

[32] Vankova Z., Peterka O., Chocholouskova M., Wolrab D., Jirasko R., Holcapek M. (2022). Retention dependences support highly confident identification of lipid species in human plasma by reversed-phase UHPLC/MS. Anal. Bioanal. Chem..

[33] O'Donnell V.B., Schebb N.H., Milne G.L., Murphy M.P., Thomas C.P., Steinhilber D. (2023). Failure to apply standard limit-of-detection or limit-of-quantitation criteria to specialized pro-resolving mediator analysis incorrectly characterizes their presence in biological samples. Nat. Commun..

[34] Höring M., Ejsing C.S., Hermansson M., Liebisch G. (2019). Quantification of cholesterol and cholesteryl ester by direct flow injection high-resolution fourier transform mass spectrometry utilizing species-specific response factors. Anal. Chem..

[35] Troppmair N., Kopczynski D., Assinger A., Lehmann R., Coman C., Ahrends R. (2023). Accurate sphingolipid quantification reducing fragmentation bias by Nonlinear models. Anal. Chem..

[36] Liebisch G., Lieser B., Rathenberg J., Drobnik W., Schmitz G. (2004). High-throughput quantification of phosphatidylcholine and sphingomyelin by electrospray ionization tandem mass spectrometry coupled with isotope correction algorithm. Biochim. Biophys. Acta.

[37] Schuhmann K., Moon H., Thomas H., Ackerman J.M., Groessl M., Wagner N. (2019). Quantitative fragmentation model for bottom-up shotgun lipidomics. Anal. Chem..

[38] Canez C.R., Li L. (2024). Studies of labware contamination during lipid extraction in mass spectrometry-based lipidome analysis. Anal. Chem..

[39] Heiskanen L.A., Suoniemi M., Ta H.X., Tarasov K., Ekroos K. (2013). Long-term performance and stability of molecular shotgun lipidomic analysis of human plasma samples. Anal. Chem..

[40] Haug K., Salek R.M., Conesa P., Hastings J., de Matos P., Rijnbeek M. (2012). MetaboLights—an open-access general-purpose repository for metabolomics studies and associated meta-data. Nucleic Acids Res..

[41] Sud M., Fahy E., Cotter D., Azam K., Vadivelu I., Burant C. (2016). Metabolomics Workbench: an international repository for metabolomics data and metadata, metabolite standards, protocols, tutorials and training, and analysis tools. Nucleic Acids Res..

[42] Hermjakob H., Apweiler R. (2006). The proteomics identifications database (PRIDE) and the ProteomExchange consortium: making proteomics data accessible. Expert Rev. Proteomics.

[43] Hoffmann N., Rein J., Sachsenberg T., Hartler J., Haug K., Mayer G. (2019). mzTab-M: a data standard for sharing quantitative results in mass spectrometry metabolomics. Anal. Chem..

[44] Deutsch E.W., Vizcaíno J.A., Jones A.R., Binz P.A., Lam H., Klein J. (2023). Proteomics standards initiative at twenty years: current activities and future work. J. Proteome Res..

[45] Field D., Sterk P., Kottmann R., De Smet J.W., Amaral-Zettler L., Cochrane G. (2014). Genomic standards consortium projects. Stand Genomic Sci..

[46] Sud M., Fahy E., Cotter D., Brown A., Dennis E.A., Glass C.K. (2007). LMSD: LIPID MAPS structure database. Nucleic Acids Res..

