# Supplementary material for: The lipidomics reporting checklist a framework for transparency of lipidomic experiments and repurposing resource data
Source: J Lipid Res. 2024 Aug 14;65(9):100621. doi: 10.1016/j.jlr.2024.100621 (PMC11417233; doi:10.1016/j.jlr.2024.100621)
Supplement: Liver FIA-QQQ.pdf [file mmc1.pdf]

Contents of Report

Created by https://lipidomicstandards.org, version v2.4.0

Direct Infusion Workflow 1
Overall study design . . . 1
Lipid extraction . . . 1
Analytical platform . . . 1
Quality control . . . 1
Method qualification and validation . . . 2
Reporting . . . 2

Sample Descriptions 2
Liver Mouse / Mouse / Tissues (e.g., liver, heart, brain) . . . 2

Lipid Class Descriptions 3
1) PE[M+H]+ / Lipid identification . . . 3
1) PE[M+H]+ / Lipid quantification . . . 3
2) PC[M+H]+ / Lipid identification . . . 4
2) PC[M+H]+ / Lipid quantification . . . 4
3) SM[M+H]+ / Lipid identification . . . 5
3) SM[M+H]+ / Lipid quantification . . . 5

Direct Infusion Workflow

Overall study design

Title of the study	Mouse Liver FIA-QQQ		
Document creation date	07/10/2024	Corresponding Email	Forename.Surname@gmail.com
Principal investigator	Forename Surname	Is the workflow targeted or untargeted?	Untargeted
Institution	University XY	Clinical	No

Lipid extraction

Extraction method	2-phase system	Were internal standards added prior extraction?	Yes
pH adjustment	None	Special conditions	-
2-phase system	MTBE	Derivatization	-

Analytical platform

Ionizaton additives	Ammonium acetate	Mass window for precursor ion isolation (in Da total isolation window)	0.8
Detector	Mass spectrometer	Mass resolution for detected ion at MS2	Low resolution
MS type	QQQ	Resolution at MS2	Low
MS vendor	Mass Spec Company	Recording mode of raw data at MS2	Profile mode
Direct type	FIA	Was/Were additional dimension/techniques used	No
MS Level	MS2		

Quality control

Blanks	Yes	Quality control	Yes
Type of Blanks	Solvent blank, Internal standard blank	Type of QC sample	Sample pool

Method qualification and validation

Method validation	Yes	Precision	Yes
Lipid recovery	Yes	Accuracy	Yes
Dynamic quantification range	Yes	Guidelines followed	None
Limit of quantitation (LOQ)/Limit of detection (LOD)	Yes		

Reporting

Are reported raw data uploaded into repository?	Available on request	Raw data upload	No
Are metadata available?	Available on request	Additional comments	-

Sample Descriptions

Liver Mouse / Mouse / Tissues (e.g., liver, heart, brain)

Perfusion	Yes	Storage time (month)	12
Storage and collection	Available	Freeze-thaw cycles	1
Provided preanalytical information	Time to freeze (min), Storage time (month), Freeze-thaw cycles	Additives	None
Temperature handling original sample	Room temperature	Were samples stored under inert gas?	No
Instant sample preparation	No	Additional preservation methods	No
Time to freeze (min)	15	Biobank samples	No
Snap freezing in liquid N2	Yes	Sample homogenization	Yes
Storage temperature	-80 °C	Sample homogenization solvent	Isopropanol

Lipid Class Descriptions

1) PE[M+H]+ / Lipid identification

Lipid class	PE	Which assumptions were presumed?	Presence of acyl-bond for all species
MS Level for identification	MS2	Check on:	Isomeric overlap, Isobaric overlap
Identification level	Species level	Limit of detection	Signal theshold
Polarity mode	Positive	Additional dimension/techniques	-
Type of positive (precursor)ion	[M+H]+	Lipid Identification Software	Homemade
Fragments for identification		Data manipulation	Smoothing, Centroiding

Fragment name
-HG(PE,141)

Isotope correction at MS2	Type 2	Nomenclature for intact lipid molecule	Yes
MS2 verified by standard	Yes	Nomenclature for fragment ions	N/A
Background check at MS2	Yes	Further identification remarks	-
Did you presume assumptions for identification?	Yes		

1) PE[M+H]+ / Lipid quantification

Quantitative	Yes	Type I isotope correction	Yes
MS Level for quantification	MS2	Limit of quantification	Signal theshold
Internal lipid standard(s) MS2		Normalization to reference	No

Internal standard	Fragment(s)	Endogenous subclass
PE 15:0/18:1[D7]	-HG(PE,141)	all PE species

Type of quantification	Calibration line	Lipid Quantification Software	Homemade
Type of calibration line	Matrix based	Batch correction	No
Species calibration line	PE 16:0/18:1, PE 18:1/18:1, PE 18:0/20:4	Further quantification remarks	-
Response correction	No		

2) PC[M+H]+ / Lipid identification

Lipid class	PC	Which assumptions were presumed?	Presence of even acyl-chains only; odd chain annotation as PC O
MS Level for identification	MS2	Check on:	Isomeric overlap, Isobaric overlap
Identification level	Species level	Limit of detection	Signal theshold
Polarity mode	Positive	Additional dimension/techniques	-
Type of positive (precursor)ion	[M+H]+	Lipid Identification Software	Homemade
Fragments for identification		Data manipulation	Smoothing, Centroiding

Fragment name
HG(PC,184)

Isotope correction at MS2	Type 2	Nomenclature for intact lipid molecule	Yes
MS2 verified by standard	Yes	Nomenclature for fragment ions	N/A
Background check at MS2	Yes	Further identification remarks	Type-II correction includes both PC and SM
Did you presume assumptions for identification?	Yes		

2) PC[M+H]+ / Lipid quantification

Quantitative	Yes	Type I isotope correction	Yes
MS Level for quantification	MS2	Limit of quantification	Signal theshold
Internal lipid standard(s) MS2		Normalization to reference	Yes

Internal standard	Fragment(s)	Endogenous subclass
PC 15:0/18:1[D7]	HG(PC,184)	all PC species

Type of quantification	Calibration line	Lipid Quantification Software	Homemade
Type of calibration line	Matrix based	Batch correction	No
Species calibration line	PC 16:0/18:1, PC 18:1/18:1, PC 18:0/20:4	Further quantification remarks	-
Response correction	No		

3) SM[M+H]+ / Lipid identification

Lipid class	SM	Which assumptions were presumed?	Presence of two OH-groups for all species
MS Level for identification	MS2	Check on:	Isomeric overlap, Isobaric overlap
Identification level	Species level	Limit of detection	Signal theshold
Polarity mode	Positive	Additional dimension/techniques	-
Type of positive (precursor)ion	[M+H]+	Lipid Identification Software	Homemade
Fragments for identification		Data manipulation	Smoothing, Centroiding
Isotope correction at MS2	Type 2	Nomenclature for intact lipid molecule	Yes
MS2 verified by standard	Yes	Nomenclature for fragment ions	N/A
Background check at MS2	Yes	Further identification remarks	Type-II correction includes both PC and SM
Did you presume assumptions for identification?	Yes		

Fragments for identification:

Fragment name
HG(PC,184)

3) SM[M+H]+ / Lipid quantification

Quantitative	Yes	Type I isotope correction	Yes
MS Level for quantification	MS2	Limit of quantification	Signal theshold
Internal lipid standard(s) MS2		Normalization to reference	Yes
Type of quantification	Calibration line	Lipid Quantification Software	Homemade
Type of calibration line	Matrix based	Batch correction	No
Species calibration line	SM 18:1;O2/16:0, SM 18:1;O2/18:1, SM 18:1;O2/24:0	Further quantification remarks	-
Response correction	No		

Internal lipid standard(s) MS2:

Internal standard	Fragment(s)	Endogenous subclass
SM 18:1;O2/12:0	HG(PC,184)	all SM species
