# Supplementary material for: The lipidomics reporting checklist a framework for transparency of lipidomic experiments and repurposing resource data
Source: J Lipid Res. 2024 Aug 14;65(9):100621. doi: 10.1016/j.jlr.2024.100621 (PMC11417233; doi:10.1016/j.jlr.2024.100621)
Supplement: Fibros HILIC-QQQ.pdf [file mmc2.pdf]

Contents of Report

Created by https://lipidomicstandards.org, version v2.4.0

Separation Workflow 1
Overall study design . . . 1
Lipid extraction . . . 1
Analytical platform . . . 1
Quality control . . . 1
Method qualification and validation . . . 2
Reporting . . . 2

Sample Descriptions 2
Fibroblasts / Human / Cells . . . 2

Lipid Class Descriptions 3
1) BMP[M+NH4]+ / Lipid identification . . . 3
1) BMP[M+NH4]+ / Lipid quantification . . . 3
2) PG[M+NH4]+ / Lipid identification . . . 4
2) PG[M+NH4]+ / Lipid quantification . . . 4

Separation Workflow

Overall study design

Title of the study	Fibroblast HILIC-QQQ		
Document creation date	07/10/2024	Corresponding Email	Forename.Surname@gmail.com
Principal investigator	Forename Surname	Is the workflow targeted or untargeted?	Targeted
Institution	University XY	Clinical	No

Lipid extraction

Extraction method	2-phase system	Were internal standards added prior extraction?	Yes
pH adjustment	None	Special conditions	-
2-phase system	Bligh&Dyer	Derivatization	-

Analytical platform

Ionizaton additives	Ammonium formate, Formic acid	Ion source	ESI
Number of separation dimensions	One dimension	MS Level	MS2
Separation type 1	LC	Mass window for precursor ion isolation (in Da total isolation window)	0.8
Separation mode 1 (liquid)	HILIC	Mass resolution for detected ion at MS2	Low resolution
Detector	Mass spectrometer	Resolution at MS2	Low
MS type	QQQ	Recording mode of raw data at MS2	Centroid mode
MS vendor	Mass Spec Company	Was/Were additional dimension/techniques used	No

Quality control

Blanks	Yes	Quality control	Yes
Type of Blanks	Extraction blank, Solvent blank, Internal standard blank	Type of QC sample	Sample pool

Method qualification and validation

Method validation	Yes	Precision	Yes
Lipid recovery	Yes	Accuracy	Yes
Dynamic quantification range	Yes	Guidelines followed	None
Limit of quantitation (LOQ)/Limit of detection (LOD)	Yes		

Reporting

Are reported raw data uploaded into repository?	Available on request	Raw data upload	No
Are metadata available?	Available on request	Additional comments	-

Sample Descriptions

Fibroblasts / Human / Cells

Storage and collection conditions	Available	Storage time (month)	1
Provided preanalytical information	Time to freeze (min), Storage time (month), Freeze-thaw cycles	Freeze-thaw cycles	1
Temperature handling original sample	Room temperature	Additives	None
Instant sample preparation	No	Were samples stored under inert gas?	No
Time to freeze (min)	15	Additional preservation methods	No
Snap freezing in liquid N2	No	Biobank samples	No
Storage temperature	-20 °C		

Lipid Class Descriptions

1) BMP[M+NH4]+ / Lipid identification

Lipid class	BMP	Limit of detection	S/N ratio > 3
MS Level for identification	MS2	RT verified by standard	Yes
Identification level	Molecular species level	Separation of isobaric/isomeric interferece confirmed	Yes
Polarity mode	Positive	Model for separation prediction	No
Type of positive (precursor)ion	[M+NH4]+	Additional dimension/techniques	-
Fragments for identification		Lipid Identification Software	Homemade

Fragment name
FA1(+C3H6O2)

Isotope correction at MS2	Type 2	Data manipulation	-
MS2 verified by standard	Yes	Nomenclature for intact lipid molecule	Yes
Background check at MS2	Yes	Nomenclature for fragment ions	N/A
Did you presume assumptions for identification?	No	Further identification remarks	Separation of isomeric PG and BMP verified with standards
Check on:	Isomeric overlap		

1) BMP[M+NH4]+ / Lipid quantification

Quantitative	Yes	Limit of quantification	No
MS Level for quantification	MS2	Normalization to reference	Yes
Internal lipid standard(s) MS2		Lipid Quantification Software	Homemade

Internal standard	Fragment(s)	Endogenous subclass
BMP 14:0/14:0	FA1(+C3H6O2)	for all BMP

Type of quantification	Internal standard amount	Batch correction	No
Response correction	No	Further quantification remarks	-
Type I isotope correction	Yes		

2) PG[M+NH4]+ / Lipid identification

Lipid class	PG	Limit of detection	S/N ratio > 3
MS Level for identification	MS2	RT verified by standard	Yes
Identification level	Species level	Separation of isobaric/isomeric interferece confirmed	Yes
Polarity mode	Positive	Model for separation prediction	No
Type of positive (precursor)ion	[M+NH4]+	Additional dimension/techniques	-
Fragments for identification		Lipid Identification Software	Homemade

Fragment name
-HG(PG,172+NH4)

Isotope correction at MS2	Type 2	Data manipulation	-
MS2 verified by standard	Yes	Nomenclature for intact lipid molecule	Yes
Background check at MS2	Yes	Nomenclature for fragment ions	N/A
Did you presume assumptions for identification?	No	Further identification remarks	Separation of isomeric PG and BMP verified with standards
Check on:	Isomeric overlap		

2) PG[M+NH4]+ / Lipid quantification

Quantitative	Yes	Limit of quantification	No
MS Level for quantification	MS2	Normalization to reference	Yes
Internal lipid standard(s) MS2		Lipid Quantification Software	Homemade

Internal standard	Fragment(s)	Endogenous subclass
PG 14:0/14:0	-HG(PG,172+NH4)	for all PG

Type of quantification	Internal standard amount	Batch correction	No
Response correction	No	Further quantification remarks	-
Type I isotope correction	Yes		
