# Supplementary material for: The lipidomics reporting checklist a framework for transparency of lipidomic experiments and repurposing resource data
Source: J Lipid Res. 2024 Aug 14;65(9):100621. doi: 10.1016/j.jlr.2024.100621 (PMC11417233; doi:10.1016/j.jlr.2024.100621)
Supplement: Plasma nano-infusion-FTMS.pdf [file mmc3.pdf]

Contents of Report

Created by https://lipidomicstandards.org, version v2.4.0

Direct Infusion Workflow 1
Overall study design 1
Lipid extraction 1
Analytical platform 1
Quality control 1
Method qualification and validation 2
Reporting 2

Sample Descriptions 2
Plasma Preanalytics Known / Human / Plasma 2
Plasma Preanalytics Unknown / Human / Plasma 2

Lipid Class Descriptions 3
1) TG[M+NH4]+ / Lipid identification 3
1) TG[M+NH4]+ / Lipid quantification 3
2) CE[M+NH4]+ / Lipid identification 3
2) CE[M+NH4]+ / Lipid quantification 4
3) PC[M+HCOO]- / Lipid identification 4
3) PC[M+HCOO]- / Lipid quantification 4
4) SM[M+HCOO]- / Lipid identification 5
4) SM[M+HCOO]- / Lipid quantification 5

Direct Infusion Workflow

Overall study design

Title of the study	Plasma nano-infusion-FTMS		
Document creation date	07/10/2024	Corresponding Email	Forename.Surname@gmail.com
Principal investigator	Forename Surname	Is the workflow targeted or untargeted?	Untargeted
Institution	University XY	Clinical	No

Lipid extraction

Extraction method	2-phase system	Were internal standards added prior extraction?	Yes
pH adjustment	None	Special conditions	-
2-phase system	MTBE	Derivatization	-

Analytical platform

Ionizaton additives	Ammonium formate	Mass resolution for detected ion at MS1	High resolution
Detector	Mass spectrometer	Resolution at m/z 200 at MS1	140000
MS type	Orbitrap	Mass accuracy in ppm at MS1	3
MS vendor	Thermo	Recording mode of raw data at MS1	Profile mode
Direct type	Chip	Was/Were additional dimension/techniques used	No
MS Level	MS1		

Quality control

Blanks	Yes	Quality control	Yes
Type of Blanks	Extraction blank, Solvent blank, Internal standard blank	Type of QC sample	Sample pool, Reference material

Method qualification and validation

Method validation	Yes	Precision	Yes
Lipid recovery	Yes	Accuracy	Yes
Dynamic quantification range	Yes	Guidelines followed	None
Limit of quantitation (LOQ)/Limit of detection (LOD)	Yes		

Reporting

Are reported raw data uploaded into repository?	Available on request	Raw data upload	No
Are metadata available?	Available on request	Additional comments	-

Sample Descriptions

Plasma Preanalytics Known / Human / Plasma

Storage and collection conditions	Available	Storage temperature	Liquid nitrogen
Provided preanalytical information	Time to separate plasma/serum (min), Time to freeze (min), Storage time (month), Freeze-thaw cycles	Storage time (month)	36
Time to separate plasma/serum (min)	30	Freeze-thaw cycles	1
Temperature handling original sample	4-8 °C	Additives	None
Instant sample preparation	No	Were samples stored under inert gas?	No
Time to freeze (min)	45	Additional preservation methods	No
Snap freezing in liquid N2	No	Biobank samples	Yes

Plasma Preanalytics Unknown / Human / Plasma

Storage and collection conditions	Unknown

Lipid Class Descriptions

1) TG[M+NH4]+ / Lipid identification

Lipid class	TG	Did you presume assumptions for identification?	No
MS Level for identification	MS1	Check on:	Isomeric overlap, Isobaric overlap
Identification level	Species level	Limit of detection	Signal theshold
Polarity mode	Positive	Additional dimension/techniques	-
Type of positive (precursor)ion	[M+NH4]+	Lipid Identification Software	ALEX
Isotope correction at MS1	No	Data manipulation	Centroiding, Lock mass correction
MS1 verified by standard	Yes	Nomenclature for intact lipid molecule	Yes
Background check at MS1	Yes	Further identification remarks	-

1) TG[M+NH4]+ / Lipid quantification

Quantitative	Yes	Limit of quantification	Accuracy and coefficient of variation (< 20 %)
MS Level for quantification	MS1	Normalization to reference	Yes
Internal lipid standard(s) MS1		Lipid Quantification Software	Homemade
Type of quantification	Internal standard amount	Batch correction	No
Response correction	No	Further quantification remarks	-
Type I isotope correction	Yes		

Internal standard	Endogenous subclass
TG 51:0	TG

2) CE[M+NH4]+ / Lipid identification

Lipid class	CE	Did you presume assumptions for identification?	No
MS Level for identification	MS1	Check on:	Isomeric overlap, Isobaric overlap, In-source fragmentation
Identification level	Species level	Limit of detection	Signal theshold
Polarity mode	Positive	Additional dimension/techniques	-
Type of positive (precursor)ion	[M+NH4]+	Lipid Identification Software	ALEX
Isotope correction at MS1	No	Data manipulation	Centroiding, Lock mass correction
MS1 verified by standard	Yes	Nomenclature for intact lipid molecule	Yes
Background check at MS1	Yes	Further identification remarks	-

2) CE[M+NH4]+ / Lipid quantification

Quantitative	Yes	Limit of quantification	Accuracy and coefficient of variation (< 20 %)
MS Level for quantification	MS1	Normalization to reference	No
Internal lipid standard(s) MS1		Lipid Quantification Software	Homemade
Type of quantification	Internal standard amount	Batch correction	No
Response correction	Response model	Further quantification remarks	-
Type I isotope correction	Yes		

Internal standard	Endogenous subclass
CE 17:0	CE

3) PC[M+HCOO]- / Lipid identification

Lipid class	PC	Which assumptions were presumed?	Only even chain fatty acyl chains
MS Level for identification	MS1	Check on:	Isomeric overlap, Isobaric overlap
Identification level	Species level	Limit of detection	Signal theshold
Polarity mode	Negative	Additional dimension/techniques	-
Type of negative (precursor)ion	[M+HCOO]-	Lipid Identification Software	ALEX
Isotope correction at MS1	No	Data manipulation	Centroiding, Lock mass correction
MS1 verified by standard	Yes	Nomenclature for intact lipid molecule	Yes
Background check at MS1	Yes	Further identification remarks	Confirmation by positive ion mode and MS2
Did you presume assumptions for identification?	Yes		

3) PC[M+HCOO]- / Lipid quantification

Quantitative	Yes	Limit of quantification	Accuracy and coefficient of variation (< 20 %)
MS Level for quantification	MS1	Normalization to reference	Yes
Internal lipid standard(s) MS1		Lipid Quantification Software	Homemade
Type of quantification	Internal standard amount	Batch correction	No
Response correction	No	Further quantification remarks	-
Type I isotope correction	Yes		

Internal standard	Endogenous subclass
PC 28:0	PC

4) SM[M+HCOO]- / Lipid identification

Lipid class	SM	Did you presume assumptions for identification?	No
MS Level for identification	MS1	Check on:	Isomeric overlap, Isobaric overlap
Identification level	Species level	Limit of detection	Signal theshold
Polarity mode	Negative	Additional dimension/techniques	-
Type of negative (precursor)ion	[M+HCOO]-	Lipid Identification Software	ALEX
Isotope correction at MS1	No	Data manipulation	Centroiding, Lock mass correction
MS1 verified by standard	Yes	Nomenclature for intact lipid molecule	Yes
Background check at MS1	Yes	Further identification remarks	Confirmation by positive ion mode and MS2

4) SM[M+HCOO]- / Lipid quantification

Quantitative	Yes	Limit of quantification	Accuracy and coefficient of variation (< 20 %)
MS Level for quantification	MS1	Normalization to reference	Yes
Internal lipid standard(s) MS1		Lipid Quantification Software	Homemade
Type of quantification	Internal standard amount	Batch correction	No
Response correction	No	Further quantification remarks	-
Type I isotope correction	Yes		

Internal standard	Endogenous subclass
SM 30:1;O2	SM
